# Current and Emerging Methods for Ovarian Cancer Screening and Diagnostics: A Comprehensive Review

**DOI:** 10.3390/cancers14122885

**Published:** 2022-06-11

**Authors:** Juliane M. Liberto, Sheng-Yin Chen, Ie-Ming Shih, Tza-Huei Wang, Tian-Li Wang, Thomas R. Pisanic

**Affiliations:** 1 Department of Pathology, Johns Hopkins University School of Medicine, Baltimore, MD 21231, USA; juliane.liberto@jhmi.edu (J.M.L.); ishih@jhmi.edu (I.-M.S.); tlw@jhmi.edu (T.-L.W.); 2 School of Medicine, Chang Gung University, 33302 Taoyuan, Taiwan; b0402025@cgu.edu.tw; 3 Department of Gynecology and Obstetrics, Johns Hopkins University School of Medicine, Baltimore, MD 21231, USA; 4 Department of Oncology, Johns Hopkins University School of Medicine, Baltimore, MD 21231, USA; thwang@jhu.edu; 5 Department of Mechanical Engineering, Johns Hopkins University, Baltimore, MD 21218, USA; 6 Johns Hopkins Institute for NanoBioTechnology, Johns Hopkins University, Baltimore, MD 21218, USA

**Keywords:** ovarian cancer, diagnostic, emerging, screening, biomarkers, HGSC

## Abstract

**Simple Summary:**

Ovarian high-grade serous carcinoma (HGSC) has a 5-year survival rate of less than 50%, making it one of the most lethal gynecological cancers for women in the developed world today. Delayed presentation of clinical symptoms and late-stage diagnosis drive the high mortality rate of this disease. Early detection is associated with significant improvements in survival, however, screening in the general population is currently not recommended at this time due to a notable lack of sensitive and specific biomarkers for early-stage disease. In this review, we provide an overview of the current landscape of ovarian cancer diagnostics, emphasizing emerging methodologies for the non-invasive detection of HGSC.

**Abstract:**

With a 5-year survival rate of less than 50%, ovarian high-grade serous carcinoma (HGSC) is one of the most highly aggressive gynecological malignancies affecting women today. The high mortality rate of HGSC is largely attributable to delays in diagnosis, as most patients remain undiagnosed until the late stages of -disease. There are currently no recommended screening tests for ovarian cancer and there thus remains an urgent need for new diagnostic methods, particularly those that can detect the disease at early stages when clinical intervention remains effective. While diagnostics for ovarian cancer share many of the same technical hurdles as for other cancer types, the low prevalence of the disease in the general population, coupled with a notable lack of sensitive and specific biomarkers, have made the development of a clinically useful screening strategy particularly challenging. Here, we present a detailed review of the overall landscape of ovarian cancer diagnostics, with emphasis on emerging methods that employ novel protein, genetic, epigenetic and imaging-based biomarkers and/or advanced diagnostic technologies for the noninvasive detection of HGSC, particularly in women at high risk due to germline mutations such as *BRCA1/2*. Lastly, we discuss the translational potential of these approaches for achieving a clinically implementable solution for screening and diagnostics of early-stage ovarian cancer as a means of ultimately improving patient outcomes in both the general and high-risk populations.

## 1. Introduction

The term “ovarian cancer” (OC) encompasses a heterogeneous group of carcinomas that form in ovarian tissue, and which present with distinct clinicopathological and molecular features along with unique tissue origins [1]. Among them, ovarian high-grade serous carcinoma (HGSC) accounts for approximately 70–80% of all ovarian cancer diagnoses [2,3] and is the most lethal gynecological malignancy affecting women in the developed world today. According to the American Cancer Society, about 19,800 new cases of OC will be diagnosed in the U.S. and an estimated 12,810 women will succumb to the disease in 2022 [4]. The high mortality rate of HGSC can be largely attributed to the difficulty in detecting the disease, particularly at the early disease stages when clinical interventions are most effective. Symptoms for HGSC are generally insidious, with little to no clinical presentation until advanced stages, when women will generally present with non-specific symptoms such as bloating, pelvic or abdominal pain, or menstrual irregularities [5]. Indeed, researchers have attempted to generate diagnostic models of ovarian cancer symptoms in order to improve OC detection, and while it was reported that symptoms are present at each stage of the disease, they were shown to be non-specific [6]. As a result, 70% of HGSC cases remain undiagnosed until FIGO stage III-IV [5,7]. Consequently, the long-term survival rate for women diagnosed with ovarian cancer remains low, with a 5-year survival rate of only 49% [8,9].

**Figure 1:**
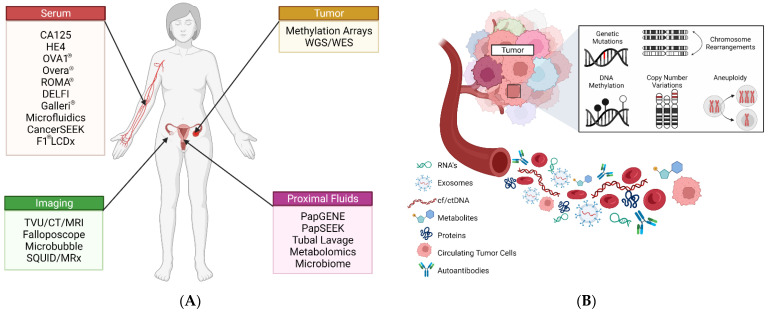
Overview of ovarian cancer screening and diagnostics. (**A**) Relevant emerging strategies for screening and detection of HGSC. (**B**) Circulating tumor biomarkers that can be assayed for screening and diagnostic purposes.

Routine screening for early detection has shown considerable benefit for many forms of cancer. For example, since the introduction of the Papanicolaou (Pap) test, the incidence and mortality of cervical cancer in the U.S. screened populations have declined by over 75% [10]. Similarly, colonoscopy screening was shown to be associated with a 70% mortality risk reduction for colorectal cancer [11]. Unfortunately, in the case of HGSC, effective screening remains unavailable, as there is “currently no strategy for early detection screening that reduces mortality or incidence of ovarian cancer” [12]. As a consequence, routine screening for HGSC is, at present, not recommended by the United States Preventative Services Task Force (USPSTF) due to the fact that “the potential harms outweigh the potential benefits” [12,13]. This decision is largely driven by the fact that the low prevalence of the disease in the general population would ostensibly result in high rates of false positivity from suboptimal tests, potentially leading to both physical, in the form of unnecessary surgical interventions, and psychological harm to women who do not have ovarian cancer [14]. Despite this fact, the clinical need for accurate screening and diagnostic tests for OC remains urgent. Likewise, numerous clinicians and researchers alike have endeavored to develop novel approaches for identifying early-stage disease from a variety of different biomarker sources (Figure 1).

While HGSC is the most common subtype of ovarian cancer and the primary focus of this review, it is important to reiterate that OC comprises a diverse group of diseases represented by multiple subtypes: low-grade serous, mucinous, endometroid, clear cell, and seromucinous carcinomas. These subtypes exhibit different clinical presentations, pathologies, morphologies, and molecular genotypes [15,16], which introduce additional challenges for developing early-stage diagnostic and screening tests. While not extensively discussed in this review, we direct the reader to several publications that have comprehensively detailed the biology and etiology of these subtypes [17,18,19,20].

## 2. Prologue—Early Screening and Risk Assessment Considerations

The terms screening and diagnostics are oftentimes used interchangeably, however, there exists an important distinction between the two in regard to both their intended clinical use and required performance characteristics. A screening test is used to assess an individual’s risk of developing a disease, and is generally targeted to large numbers of asymptomatic individuals but could also be specifically targeted to at-risk populations [21]. Conversely, a diagnostic test is used to determine whether or not an individual has a given disease, and is targeted to individuals who are symptomatic [21]. As such, a diagnostic test must be extremely accurate in determining disease, with particular emphasis on achieving high specificity for disease diagnosis [21]. Alternatively, screening tests should generally be focused on achieving high sensitivity for detecting disease in order to limit the number of false negatives leading to missed diagnoses in the test population [21]. To give an example of this distinction, the Pap test is a widely used screening method for detecting the presence of abnormal cervical cells [22], however, the occurrence of abnormal cells does not always indicate the presence of cancer. Abnormal cells could be caused by a number of factors such as mild inflammation, bacterial, viral, or yeast infections, or cervical dysplasia [23,24]. When abnormal cells are present in a Pap test, most patients are referred for colposcopy with cervical biopsy, which would serve as a highly accurate diagnostic to determine the presence of cancer [25]. Therefore, an assay may be better suited as a diagnostic or a screening test based on its clinical performance, as assessed by its clinical specificity (Spec.), clinical sensitivity (Sens.), accuracy, and negative and positive predictive values (NPV and PPV, respectively).

In general, screening and diagnostic assays are focused on detecting and/or identifying a given disease, and not necessarily predicting patient outcomes or determining appropriate treatment regimes. However, the clinical application of certain assays may go beyond the role of simply detecting disease. As such, cancer biomarkers can take on a diagnostic (capable of detecting disease), prognostic (capable of estimating patient outcomes), and/or predictive (predicting response to treatment) role [26,27]. As an example, the presence of germline or somatic *BRCA1/2* mutations is not necessarily diagnostic, as most women with these mutations do not have cancer, however, it can serve as a prognostic marker as several studies have illustrated that women with HGSC who carry a *BRCA* mutation generally have longer overall survival and progression-free survival than those who do not [27,28,29]. Additionally, *BRCA* mutation status can serve as a predictive marker, since women with homologous recombination deficiencies are known to respond well to treatments targeting this pathway such as PARP inhibitors [30,31,32]. While these terms are normally applied to single biomarkers, the clinical role of many of the multi-marker assays that are discussed in this review may additionally fall under more than one application category and could potentially be used to inform clinicians of patient outcomes in addition to being used as a screening or diagnostic test.

According to the American Cancer Society, the lifetime risk for women in the general United States population for developing OC is 1 in 78, or just under 1.3%, and the prevalence of the disease is approximately 1:2500 [33]. This low frequency implies stringent requirements are necessary for effective population-wide screening tests. For example, a given test must exhibit a sensitivity of at least 75% and a specificity greater than 99.7% in order to achieve a positive predictive value (PPV) of 10% in the general population [33]. Even with such stringent sensitivity and specificity metrics, a PPV of 10% would still imply a high number of false positives for the screening test, as for every single positive case of OC that the screening test identifies, nine other cases would be falsely identified leading to unnecessary and potentially invasive follow-up tests. The risk associated with low PPV values is perhaps best highlighted by the case of the CLIA-certified OvaSure™ screening test, which was marketed based on inaccurate estimates of PPV and had to be abruptly discontinued due to numerous cases of false-positive results [34].

In general, there are two ways to improve PPV, the first of which is to increase test specificity (fewer false positives), however, this typically results in an undesirable reduction in clinical sensitivity (fewer true positives). The other means of improving PPV is to restrict screening to high-risk populations since PPV is positively influenced by an increased frequency of disease in the test population [35]. When testing in higher-risk populations, such as women with *BRCA1/2* mutations, the test metrics can be relaxed slightly. For example, a screening test with 75% sensitivity at 98% specificity would yield a PPV of 13% in this population [33]. A challenge still remains in efficiently identifying women at an increased risk of developing HGSC that would most benefit from such screening approaches. Current approaches aimed at improving risk predictions are discussed briefly in Appendix A of this review, including a detailed discussion of factors that place women at a higher risk of developing ovarian cancer.

### Cost-Effectiveness of Ovarian Cancer Screening

In order to achieve clinical utility, early screening for any disease or malignancy must meet several basic requirements. Namely, the disease to be tested must be frequent enough in the target population to justify screening efforts, it should pose a significant risk of mortality to those who have the disease and remain untreated and, if the disease is caught early, there must be effective treatments available to patients to improve outcome [36,37]. Furthermore, screening tests should also in general be affordable, offer minimal risk, and be accessible to the appropriate patient population [36,37]. The cost-effectiveness of ovarian cancer screening in the general population has been evaluated across several different studies [38,39,40]. In a secondary analysis of the study data from the UKCTOCS clinical trial, Moss et al. illustrated that multimodal screening for women above the age of 50 is cost-effective, with the caveat that women who will be screened are willing to pay approximately USD 150,000 per quality-adjusted life years (QALY) [38]. In their model, multimodal screening costs for ovarian cancer were calculated based on the probability that a patient would undergo TVU, physician assessment, further imaging or biomarker tests, consultation with a gynecologic oncologist, and/or surgery [38]. Another study, also based on the UKCTOCS study data, estimated costs to be approximately GBP 8864 (USD 11,200) per QALY for women residing in the U.K. [39]. The lower incremental cost-effectiveness ratio (ICER) to patients for this study is likely driven by reduced public health care costs for U.K. residents compared to women living in the U.S. Havrilesky et al. used a theoretical approach to illustrate that a moderately well-performing screening test (85% sensitivity at 95% specificity) used in women between the ages of 50 and 85 could be cost-effective compared to no screening at all [40]. Their hypothetical calculations showed ICER ranges of USD 73,496/QALY for the general population to USD 36,025/QALY for high-risk individuals [40]. Regardless, the economic impact of ovarian cancer screening for patients is high, and it is clear that the need for effective screening options is necessary to reduce health care costs.

## 3. Current Screening Options

### CA125 and Transvaginal Ultrasound

**Figure 2:**
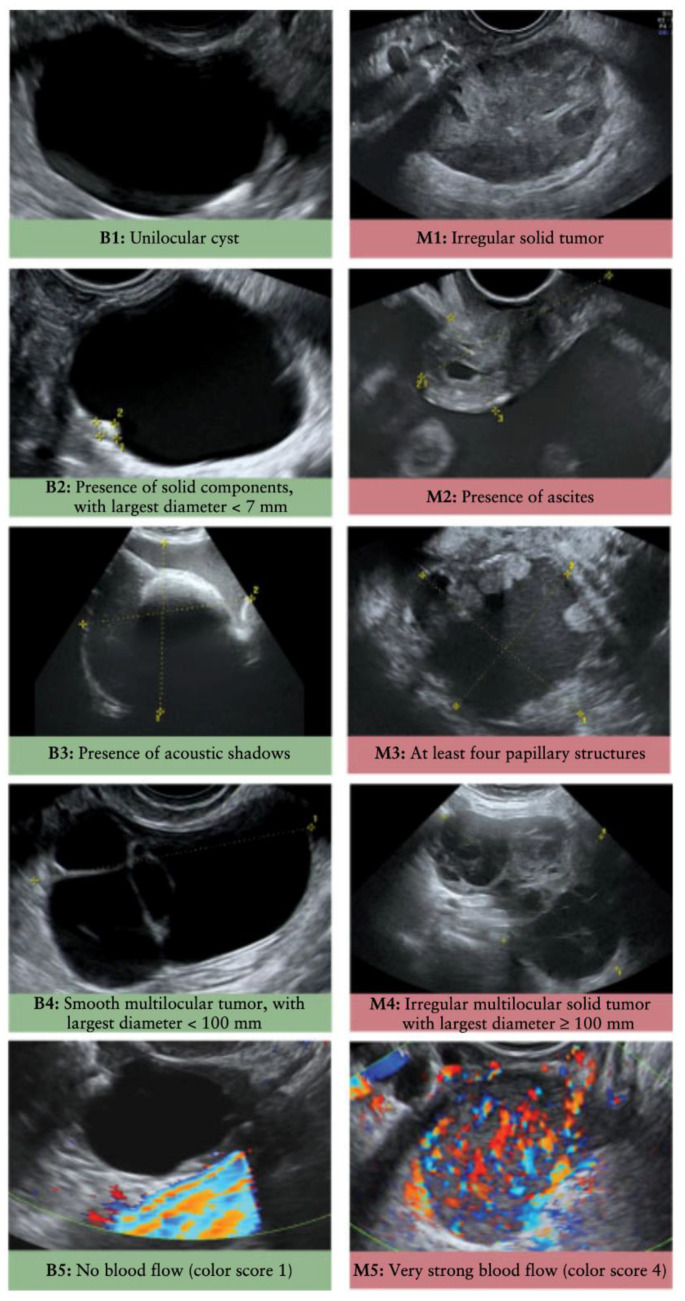
Representative sonographic images of benign (**B1**–**B5**) and malignant (**M1**–**M5**) ovarian masses. Features in each panel are reviewed based on the IOTA simple rules to evaluate malignancy of ovarian lesions. In general, the presence of irregular shaped bodies, papillary projections, and/or internal blood flow are predictive of malignancy. Images provided from Kaijser et al. [41]. Used with permission.

The current clinically available approaches for OC screening are primarily limited to physical assessment by clinicians, imaging of the adnexa by transvaginal ultrasound (TVU) (Figure 2), and serum level measurements of protein biomarker cancer antigen 125 (CA125). TVU is the most common imaging modality employed for detecting ovarian cancer and allows clinicians to identify irregularities in the size and shape of ovarian tissues. Images obtained by TVU are reviewed by radiologists to assess the presence of specific clinical features based on the International Ovarian Tumor Analysis (IOTA) simple rules. Some of the assessed features include the presence of papillary projections, ascites, and/or internal blood flow, which are ultimately used to predict the likelihood and stage of malignant masses [41].

CA125, the most widely used OC biomarker, is an epithelial cell surface glycoprotein that is implicated in promoting cancer cell growth and metastasis. Initial findings for CA125 identified elevated serum levels (>35 U/mL) of this protein in ~80% of ovarian cancer patients [42,43,44]. Unfortunately, further investigations into the clinical benefit of CA125 screening have yet to demonstrate substantial benefit to patients, primarily due to a lack of clinical sensitivity for CA125 at the early stages of disease. In the preoperative setting, CA125 levels are reported to have limited value for improved discrimination of ovarian masses when applied to multimodal diagnostic approaches [45]. Findings from the Prostate, Lung, Colorectal and Ovarian (PLCO) Screening Trial showed that screening with CA125 exhibited a positive predictive value of only 4%, which could be further improved to 26.5% with the addition of TVU [46], but this combination has still not been shown to provide a tangible improvement in survival outcomes for patients after a 15 year follow up [47]. In addition, for the 28,506 women in the PLCO Trial who had results for both CA125 and TVU, only 2% of patients showed abnormalities in both tests [46].

In 2016, Jacobs et al. published the study outcomes of one of the largest ovarian cancer randomized control trials to date, the United Kingdom Collaborative Trial of Ovarian Cancer Screening (UKCTOCS) trial [48]. This multi-center study enrolled over 200,000 post-menopausal women and followed them over an 11.1 year median time period with the aim of understanding the effect that early screening with the Risk of Ovarian Cancer Algorithm (ROCA) has on ovarian cancer mortality. ROCA takes into account baseline and serially-collected CA125 levels, patient age and menopausal status, as well as potential risk factors, such as *BRCA* status, to stratify patient risk. While the study reported improved survival in the screened populations, the results were not significant, further highlighting the limitations of CA125 [48]. The limitations of CA125 in diagnosing other OC subtypes have additionally been reported. Expression of CA125 in mucinous, endometroid and other less common subtypes of OC was found to be lower than expression levels seen in serous carcinomas [49,50], further highlighting its inadequacy as a standalone biomarker for ovarian cancer diagnosis. Furthermore, epidemiological factors such as age, race, ethnicity, and obesity were shown to be implicated in influencing CA125 serum levels, regardless of the presence of ovarian cancer [51,52]. Despite its poor performance as a screening biomarker, CA125 does show clinical benefit for patients when used as a post-operative marker for monitoring patient response to therapy and facilitates detection of recurrent disease [53,54].

Other single biomarkers that have recently gained attention for their increased expression in less common subtypes of ovarian cancer are human epididymis protein 4 (HE4) and cancer antigen 19-9 (CA19-9). HE4, similar to CA125, is a surface glycoprotein, and increased secretion of HE4 has been reported to be linked to serous and endometroid ovarian tumors, but not in clear-cell or mucinous tumors [55,56]. In comparison studies of HE4 and CA125, it was reported that changes in HE4 occur 2 to 3 months earlier than in CA125 [57,58]. In 2008, the FDA cleared serum tests measuring HE4 for monitoring women who are known to have epithelial ovarian cancer, concluding that HE4 was equivalent to CA125 in surveilling disease progression [59,60]. It is important to point out that although CA125 and HE4 are FDA cleared for monitoring recurrence, neither biomarker is approved or cleared for use as a preoperative diagnostic and should not be used without further imaging or physician assessment. Increased expression of CA19-9, a monosialoganglioside, has been linked to several tumors of mucinous histology within the gastrointestinal tract, including the pancreas, liver and gall bladder [61,62]. Additional studies have reported increased expression of CA19-9 in primary mucinous ovarian carcinomas [62,63,64,65], however, conflicting evidence of the preoperative use of this biomarker in differentiating malignant versus benign mucinous ovarian tumors was reported [63].

Discussed in the following sections of this review are emerging diagnostic and screening strategies for early-stage detection of HGSC. A highlight of select biomarkers and assays, and their potential clinical applications are summarized in Table 1 and Supplementary Tables S1–S4.

## 4. Protein Diagnostics

Cancer is often termed a genetic disease, while proteins represent the functional end products of genes that can ultimately contribute to the cancer phenotype. As such, the study of cancer proteomics may provide useful insight into understanding the true molecular mechanisms associated with cancer development. Single and multi-marker blood assays have been proposed as potential diagnostic tools for detecting ovarian malignancies. A summarized table of the protein-based assays presented below can be found in Supplemental Table S1.

### 4.1. Protein Screening and Diagnostic Assays for Asymptomatic Women

CA125 is the most important OC biomarker discovered to date [37,38,39]; however, there remains an ongoing search for alternative protein biomarkers for use in coordination or in comparison with CA125. The majority of these tests have exhibited increased accuracy for the detection of ovarian cancer over CA125 alone while maintaining high specificity. Due to the large number of publications in this area, we restricted our review to include only those methods that were validated using one or more of the following methods: hold-out validation, cross-validation, and/or bootstrap validation.

Both Skates et al. [88] and Zhang et al. [89] demonstrated that a four-marker panel consisting of CA125, CA72-4, CA15-3, and MCSF, was able to achieve significantly improved sensitivity (70%) at fixed 98% specificity, over CA125 alone (40%), when used in either a Multivariate Discriminate Analysis model [88] or an Artificial Neural Network model [89], respectively (Table 1 and Table S1). Another four-marker panel, composed of CA125, ApoA-I, TTR, and a cleavage fragment of inter α-trypsin inhibitor H4 (ITIH4), also showed increased sensitivity, from 66% to 86% at 98% specificity, when compared to CA125 [106]. Yurkovetsky et al. reported a comparable increase in sensitivity using a four-marker panel consisting of CA125, HE4, carcinoembryonic antigen (CEA), and vascular cell adhesion molecule 1 (VCAM-1), which exhibited 86% sensitivity at 98% specificity for early-stage detection [107].

Researchers from Yale University identified a six-marker panel (CA125, leptin, prolactin (PRL), osteopontinin (OPN), insulin-like growth factor 2 (IGF-II), and macrophage inhibitory factor (MIF)) that exhibited 95% sensitivity at 98% specificity for all OC stages [108] and, in a follow-up study, achieved 95% sensitivity at 95% specificity for early-stage I-II disease [109]. Translation of this panel ultimately led to the development of OvaSure™, a commercially available ‘Laboratory Developed’ blood test that was sold under CLIA certification [110]. However, a thorough review by the scientific community [34,111,112] and the FDA [113] showed that the authors overestimated the performance of their assay, resulting in an unfortunately high false-positive rate, and OvaSure™ was discontinued after only four months on the market. While much controversy surrounds OvaSure™, the proteins identified in this panel continue to be explored by other researchers. In 2022, Watrowski et al. showed that this six-marker panel combined with age (>50) illustrated an area under the receiver-operating characteristic curve (AUC) of 0.89 for identifying disease in a validation cohort, however, performance was poor (AUC < 0.6) when used in women under the age of 50 [70]. Nonetheless, two proteins from this panel, OPN and MIF, have additionally demonstrated utility by improving the detection of early-stage HGSC from 65% to 84% when combined with IL-8 autoantibodies [114].

Two multi-marker panels (CA125, HE4, glycodelin, plasminogen, PLAUR, CA15-3, PAI-1 and CA125, CA19-9, EGFR, CRP, myoglobin, ApoA-1, ApoC-III, MIP-1α, IL-6, IL-18, tenascin C) exhibited high sensitivities from 80.5% to 91.3% and specificities from 96.5% to 88.5%, respectively, for the detection of early-stage ovarian cancer [115,116]. In 2019, a panel of 11 proteins was identified and validated on a custom 11-plex proximity extension assay. Using four unique validation cohorts, the authors reported that the panel exhibited 85% sensitivity at 93% specificity for detection across all stages [117]. In a Phase 3 clinical trial, Edgell et al. reported that a five-marker panel consisting of CA125, CRP, IL-6, IL-8 and serum amyloid A (SAA), exhibited high sensitivity and specificity, 92.3% and 91.3%, respectively, for early-stage disease [118].

A new screening tool, proposed by Russell et al. in 2019, was reported to detect ovarian cancer one-to-two years earlier than current clinical methods for both Type I and Type II ovarian cancer cases. The authors assessed biomarker expression profiles from seven years of blood samples, which had been longitudinally collected as part of the UKCTOCS trial. The results of the study showed that dysregulation from baseline measurements of four previously identified protein markers (CA125, phosphatidylcholine-sterol acyltransferase (LCAT), vitamin K-dependent protein Z (PROZ), and CRP [119]), could be used to identify women that subsequently develop ovarian cancer, with an AUC of 0.848 for OC samples that were 1–2 years prior to time of diagnosis [120]. While larger prospective trials will be needed to validate its performance, the clinical utility of this screening tool would ostensibly be significant, as one-to-two-year earlier detection would likely greatly impact survival outcomes for patients by shifting the time of diagnosis of disease to more treatable stages. On the other hand, the requirement of the model for long-term (seven years) longitudinal data from annual blood draws would likely be challenging to implement in the general population.

### 4.2. Emerging Protein Diagnostics

Traditionally, serum proteins such as CA125 and many of those proteins mentioned above are quantified using analysis methods such as radiolabeled immunoassays (RIA) or enzyme-based immunoassays (ELISAs). More recently, fluorescently labeled antibody-conjugated beads in combination with flow cytometry methods were employed in a number of studies for identifying OC-specific protein levels in serum [107,108,114,115,118]. While immunoassay methods have proven to be technically robust, there are a few emerging protein techniques that have stepped away from these traditional strategies.

One recent area of interest is the potential utility of platelets for the identification and diagnosis of ovarian cancer. This interest stems from the fact that thrombocytosis, i.e., platelet counts greater than 400 × 10^9^ U/L [121], was observed in approximately 23–56% of ovarian cancer patients [122] and is significantly associated with advanced disease stages and reduced survival [123,124,125]. The phenomenon of paraneoplastic thrombocytosis observed in ovarian cancer was also linked to an increase in hepatic thrombopoietin in response to tumor-derived IL-6 production [125]. Additional data suggest that platelets are able to increase tumor expression of PD-L1 through NF-κB and TGFβR1/Smad signaling [126]. In 2016, Watrowski et al. illustrated that the combination of platelet counts (>350 × 10^9^ U/L) with CA125 levels (>35 U/mL) was able to distinguish ovarian cancer patients from benign controls with an 81% sensitivity at 94% specificity, implying that platelet count may be a suitable diagnostic method for identifying ovarian cancer, specifically in settings where limited health care options are available [122]. In another study, Swedish researchers developed a model consisting of 16 platelet proteins with altered expression in ovarian cancer [127]. Using this panel, their model could distinguish late-stage ovarian cancer from benign adnexal masses with a sensitivity of 70% at 83% specificity [127]. Despite the modest diagnostic performance, the model represented the first time that the proteome of platelets was studied and proposed for use as a diagnostic for ovarian cancer. Model refinement and larger cohort sizes comprising both early-stage and borderline tumors are likely to strengthen the diagnostic sensitivity and specificity of this model.

Gyllensten et al. employed the use of a proximity extension assay (PEA) combined with next generation sequencing (NGS) and multivariate modeling to identify an 11 plasma protein panel (ALPP, CXCL8, DPY30, IL6, IL12, KRT19, PAEP, TSPAN1, SIGLEC5, VTCN1, and WFDC2) capable of distinguishing early- from late-stage HGSC with an AUC of 0.81 when tested in an independent cohort of patients [128]. Additionally, they reported that this same model showed very promising diagnostic potential, discriminating healthy from ovarian cancer patients (stage I-IV) with a sensitivity of 75% at 100% specificity [128].

Another area of active interest has been the development of microfluidic/lab-on-a-chip-based approaches that offer real-time point-of-care (POC) results to patients. One such method, reported by the McDevitt group at New York University, utilized a programmable Bio-Nano-Chip (p-BNC) [129,130] to measure CA125, HE4, CA72-4, and matrix-metalloprotease 7 (MMP-7) protein levels in patient serum in a rapid 43-min test [131]. The test is performed by adding a few hundred microliters of patient serum to a disposable chip containing an array of antibody-bound agarose beads in a sandwich immunoassay and imaged with an epifluorescence microscope. Overall, the p-BNC chip exhibited 68.7% sensitivity at 80% specificity for discrimination of ovarian cancer from benign lesions and healthy controls [131].

Within the last 5 years, there has also been an emergence of studies reporting the use of aptamer-based systems for measuring serum CA125 as a means of achieving increased performance over traditional immunoassay methods [132,133,134,135,136]. In one study by Jin et al., the authors employed silver sulfide quantum dots (Ag_2_S QD) bound to negatively charged DNA/5-flurouracil aptamers to achieve detection of CA125 at concentrations as low as 0.07 ng/mL [135]. Chen et al. reported using an aptamer-based fluorescent sensor combined with a resonance light scatter (RLS) sensor to simultaneously measure serum CA125 and stress-induced phosphoprotein 1 (STIP1) [132], a protein previously identified as a secreted biomarker in ovarian tissue with good performance for detecting early-stage tumors [137]. Their biosensors were able to detect CA125 down to 0.05 U/mL and STIP1 down to as low as 1 ng/mL, providing excellent analytical sensitivity and specificity for these two biomarkers. Similarly, Xu et al. developed a dual-color aptasensor system for concurrent measurements of CA125 and CEA, in which analyte presence at threshold concentrations result in the aggregation of salt-induced gold nanoparticles leading to the formation of fluorescent analyte-silver nanoclusters [133]. Using this system, the authors demonstrated the ability to detect CA125 and CEA down to 0.015 U/mL and 7.5 pg/mL, respectively, in as little as 2 μL of serum [133]. Farzin et al. showed an even lower limit of detection for CA125 in human serum (0.0042 U/mL) using an alternative nanobiosensing platform [136]. Their approach utilizes “doping” an amidoxime-modified polyacrylonitrile nanofiber (PAN-oxime NF) with silver nanoparticles (AgNPs), which increase electrical conductivity for improved signal amplification. The AgNPs-PAN-oxime NF is bound to an indium tin oxide glass electrode and mixed with an aminated aptamer which hybridizes to amino groups on the PAN-oxime NF. Displacement of a signaling probe by CA125 binding to the aptamer is measured as a change in voltage which can be quantified for high sensitivity detection [136].

**Figure 3:**
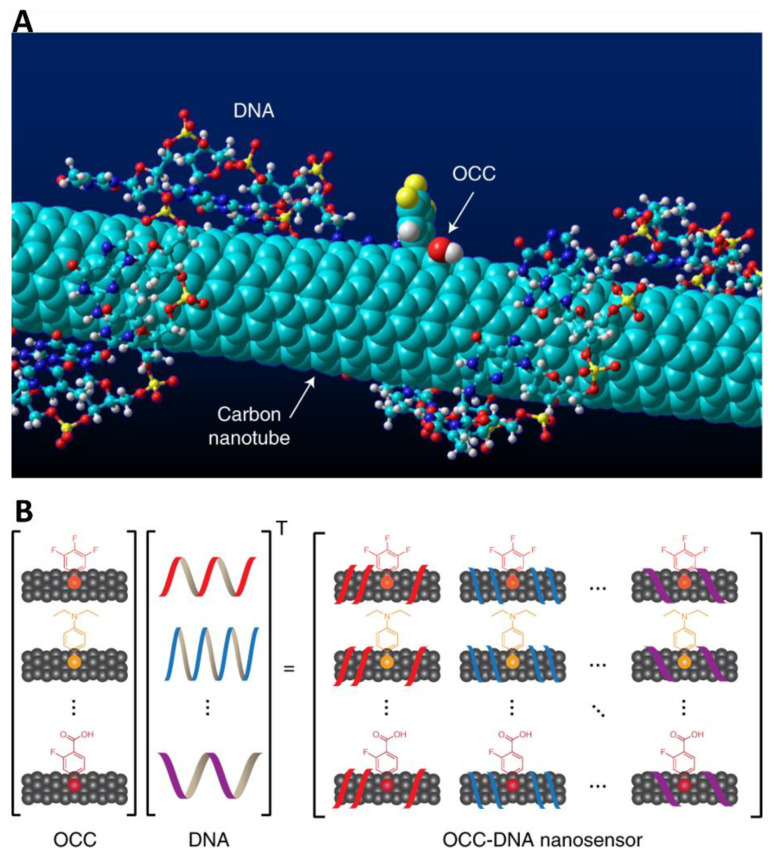
Molecular model and array construction of OCC-DNA nanosensor elements created by Kim et al. [138]. (**A**) Fabricated molecular model of a single OCC-DNA nanosensor with ssDNA wrapped around a semi-conducting single-walled nanotube (SWNCT) modified with organic color sensors (OCC). (**B**) Nanosensor array construction is generated from a matrix combination of OCC-modified SWNCTs with DNA. Used with permission.

So-called “perception-based” approaches offer an additional new tool for classifying disease states, whereby an array of non-specific responses can be combined and processed with machine learning to produce a singular response that is highly specific and accurate. In one example, Kim et al. generated a nanosensing array that consisted of modified semiconducting single-walled carbon nanotubes (SWCNTs) (Figure 3) [138]. SWCNTs are similar to Ag_2_S QD’s in that they also exhibit intrinsic near-infrared fluorescence detectable even down to the single-molecule level. Organic color centers (OCC), covalently bound molecules that enhance SWCNT photoluminescent properties, and DNA fragments were applied to the carbon nanotubes to produce an array of unique OCC-DNA sensors. A nanosensing array based on this paradigm was able to detect HGSC in serum with increased sensitivity and specificity than traditional serum and TVU-based approaches (87% sens. at 98% spec.; 84% sens. at 98% spec., respectively) [138].

**Figure 4:**
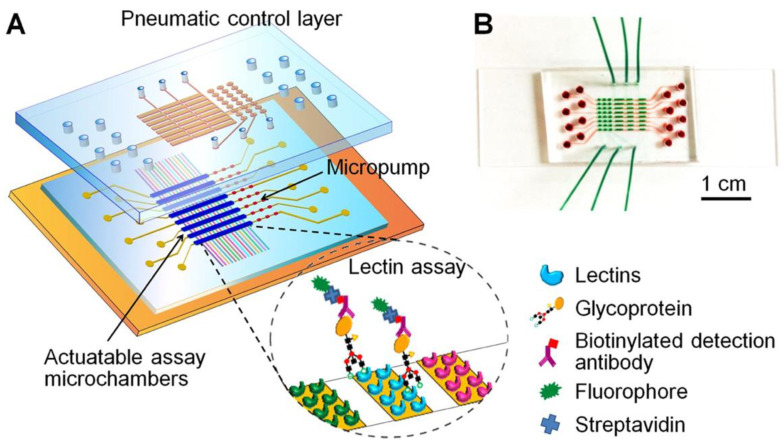
Schematic of the antibody-lectin barcode microfluidic platform proposed by Shang et al. [140]. (**A**) The integrated microfluidic lectin barcode platform contains eight identical microchambers that lay perpendicular to an array of lectins patterned on the chip’s surface. Pneumatic actuation within the assay chamber allows for mixing and capture of glycoproteins to the lectin array. (**B**) Digital image of the microchip with loading chambers in red and pneumatic control layers in green. Reprinted under the Creative Commons CC BY-4.0 license.

Glycosylation of CA125 has also recently been an area of focus since specific abnormalities in N-terminal glycosylation patterns were shown to be unique to ovarian cancer [139]. Shang et al. reported the development of a novel antibody-lectin barcode microfluidic platform for glycomic profiling of CA125 (Figure 4) [140]. The microfluidic chip is designed with eight parallel microchambers, each comprising an array of micro-barcoded lectins capable of capturing specific glycoproteins. Once captured, these glycoproteins are then tagged with biotinylated antibodies and fluorescently labeled streptavidin. After capture and tagging, the chip is imaged using an inverted fluorescent microscope and the intensity within each barcode spot is then quantified. For the lectins selected in the array chip, the best sensitivity for CA125 was seen with Concanavalin A Lectin (ConA), 0.188 U/mL, and Sambucus Nigra Lectin (SNA), 0.153 u/mL [140].

In 2020, Bayoumy et al. reported the development of a custom-built Lateral Flow Immunoassay (LFIA) platform to measure glycovariations of CA125. LFIA is an established POC assay that is simple to use and can give results in about 30 min with minimal required equipment [141]. Briefly, patient serum is applied to the sample pad and will migrate laterally down the strip towards the absorbent pad. If the serum sample contains elevated levels of glycosylated CA215 (STn-CA125) it will become sandwiched between the Anti-STn antibody located on the test line and the tracer (upconverting nanoparticles coated with Anti-CA125 monoclonal antibody). If the serum sample contains less than the detectable limit of glycosylated CA125, the tracer will only bind the control line. The strip is inserted into instrumentation that shines an infrared laser light on the detection window to excite the upconverting nanoparticles and the concentration is then quantified based on peak intensity. Using this novel method, the authors reported that the STn-CA125 LFIA assay had a 72% sensitivity at 98% specificity, which was much improved from the traditional CA125 RIA assay which showed a 16% sensitivity at 98% specificity [141].

Since its initial identification by Robert Bast in 1981 [39], researchers have employed a host of strategies based on combining multiple or individual blood protein markers with CA125 in an effort to improve its diagnostic sensitivity and specificity. While there are a number of protein biomarker panels and emerging techniques that have beenpublished with high sensitivity and specificity, especially when compared with the individual performance of CA125, adoption of these assays in the clinical setting has been slow, largely due to the lack of demonstrated survival benefit for CA125 based approaches. For many of these biomarker panels and emerging assays, larger cohort studies and clinical trials will be essential next steps to validate their clinical performance. Additionally, integrated proteomic analyses of HGSC, such as those published by the Clinical Proteomic Tumor Analysis Consortium (CPTAC) [142], can serve as significant resources for identifying new protein biomarkers that can be assayed for OC screening and diagnosis. Despite a slow transition, there appears to be an abundance of promising new innovative ideas and approaches to protein diagnostics for ovarian cancer that may bring clinical benefit in the near future.

## 5. Imaging Diagnostics

Currently, the most explored method for evaluating ovarian masses is TVU, despite the poor ability of sonographic imaging to determine malignancy, especially in patients that present with borderline and early-stage lesions. Therefore, new imaging-based approaches for ovarian cancer screening will undoubtedly be of benefit to women, as accurate determination of benign vs. malignant masses would limit unnecessary and invasive follow-up. A summarized table of the imaging-based assays presented below can be found in Supplemental Table S2.

Improvements in imaging approaches are being pursued in two major areas of research: (1) enhancement of current TVU imaging methods to improve accuracy, and (2) the development of new imaging modalities that can accurately detect malignant ovarian masses.

Doppler techniques and microbubble enhancement have been proposed as means of improving upon traditional TVU. Under Doppler ultrasound, Bedi et al. found early-stage ovarian cancer presented an abnormal central ovarian vascularity, distinct from the normal hilar or peripheral blood flow [143]. Studies further identified properties such as internal vascularity, low pulsatility indices, and low resistive indices under Doppler ultrasound were associated with ovarian malignancies [144,145]. Using a risk classification based on sonographic findings, Barroilhet et al. illustrated 89% sensitivity at 57% specificity for identifying invasive and borderline cancers through the use of Doppler-based imaging [144]. A multicenter study concluded that transvaginal color Doppler ultrasound outperformed grayscale sonography in diagnostic accuracy [146]. However, the results were not produced in a screening setting.

**Figure 5:**
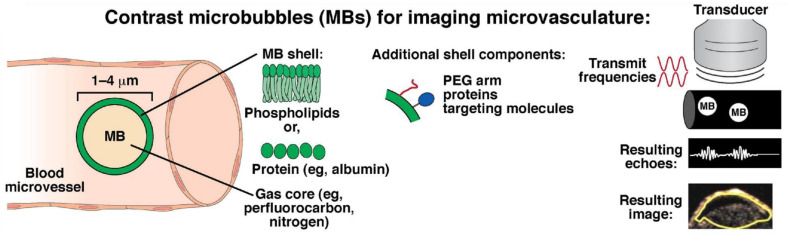
Principles of contrast-enhanced ultrasound imaging with contrast microbubble reported by Pysz et al. [147] Microbubbles generally consist of a gaseous core that is enveloped in a lipid or protein shell that may or may not be “decorated” with additional components for stability. Due to their micron size, microbubbles are able to travel within tiny microvascular spaces, including microcapillaries that may be present in solid lesions. Used with permission.

Microbubbles are microspheres with a protein or lipid shell and a gas core and are commonly used as contrast agents for ultrasound imaging (Figure 5) [147]. Microbubble contrast-enhanced TVU can be used to identify abnormally increased blood flow or tortuous vascular distribution and differentiate malignant from benign adnexal masses [148]. Nevertheless, microbubble contrast-enhanced TVU would be expected to primarily enhance the specificity of sonography but not the sensitivity for detecting early-stage disease [149].

TVU can also be integrated with photoacoustic imaging (PAI) to further improve diagnostic accuracy. The key strength of PAI is that it can collect real-time functional and molecular information from tissues without radiation or exogenous contrast [150]. PAI facilitates high-resolution detection of angiogenesis, and thus could potentially be used to detect neovascularization in early-stage OC [151,152]. However, current PAI methods are only able to penetrate tissues up to approximately 5 cm, and the spatial resolution of the technique declines with increasing depth. Therefore, TVU co-registration is required for PAI to generate more accurate structural information. Recently, a study of ex vivo detection of OC with co-registered photoacoustic and ultrasound imaging from 15 pre- and post-menopausal patients that had undergone prophylactic oophorectomy reported a sensitivity of 87.7% at a specificity of 97.9% [153]. Another in vivo study using the same technique successfully identified 26 ovarian masses and distinguished malignant cancers from benign tumors by increased tumor vascularity and decreased oxygen saturation [154].

### 5.1. Internal Organ Scans

MRI is a frequently employed secondary imaging technique that uses a magnetic field and radio waves to create a high contrast image of internal organs and tissues [155]. The use of pelvic MRI to generate high-resolution images was employed to better visualize small structures on ovarian masses [156,157] such as papillary projections, which are characteristic of epithelial tumors. Recently, the idea of applying artificial intelligence to MRI was employed. Wang et al. used a convolutional neural network model consisting of MRI data and clinical variables to distinguish OC from benign lesions with a sensitivity of 75% at a specificity of 92% [158]. In a comparative study of 103 women with adnexal masses, the diagnostic consensus of MRI to histology confirmed malignancy vs. benign lesions was comparable to TVU (MRI 83% sens. at 84% spec.; TVU 92% sens. at 59% spec.) with the conclusion that TVU should be the method of choice for initial screening, while MRI can be a useful tool for further exploration of inconclusive or indeterminate masses [159].

The combination of PET and CT scans for characterizing ovarian masses has also been proposed [159,160,161]. PET is a nuclear imaging tool that is used to look for cancer by monitoring glucose consumption in tissues by tracing cell uptake of radioactive sugar. CT on the other hand uses multiple thin X-ray images to generate a 3D image of organs and tissues. Combined, PET/CT is able to not only provide structure (from CT scan) but also function (from PET scan) of masses to clinicians. PET/CT was also shown to better identify distant lymph node metastasis when compared to other imaging modalities [162]. In a correlative study of 133 women suspected of having ovarian cancer, PET/CT imaging, when compared to pelvic ultrasound, demonstrated a 92% versus 83% accuracy, respectively, for discrimination of borderline and malignant tumors from benign masses [162]. Other work in a smaller cohort of 50 patients showed that PET/CT outperformed TVU in being able to stage carcinomas (PET/CT = 69% accuracy, TVU = 53% accuracy) [161]. However, the cystic structure of ovarian masses presents a challenge for PET/CT, and other imaging techniques, to distinguish early-stage lesions. This was further concluded in a large comparative study of TVU, MRI, and PET/CT where Grab et al. highlighted that a negative MRI or PET result does not exclude the possibility of early-stage or borderline ovarian tumors [160]. While MRI and CT/PET illustrate advances in feature characterization of adnexal lesions, TVU remains at the forefront for ovarian cancer screening due to its widely available use, low risk, and affordability.

### 5.2. Hysteroscopy Imaging

Hysteroscopy and laparoscopy approaches have been proposed as methods for early OC screening, diagnosis and can enable the collection of [potentially cancerous] endoluminal cells [163,164,165]. Cytuity™ is an FDA-cleared hysteroscopic catheter with a balloon-like mechanism on the distal end of the catheter that is able to collect tubal epithelial cells from the ostium of the fallopian tube [163]. Cells can then be sent for further cytological evaluation. In their small pilot study, Lum et al. showed that they could successfully capture endoluminal cells for cytologic analysis using either hysteroscopy or laparoscopy [165]. While the authors demonstrated that laparoscopy could be used to collect cells from both the proximal and distal end of the fallopian tube, the hysteroscopic approach was only able to capture cells from the proximal end of the tube due to the acute angle of the fallopian tube to the uterus.

Falloposcope devices have been in development since as early as 1990, and are intended to provide a means of visualizing the fallopian tubes through transvaginal endoscopy [166,167]. Early falloposcope designs were limited in the number of optical fibers that could be incorporated into a small diameter catheter [166], resulting in low-resolution images and an unacceptably high rate of missed lesions [168]. More recently, however, improvements to this technology have improved the outlook for implementation of this technique in clinical applications such as OC screening and diagnostics [168]. For example, researchers at the University of Arizona have reported the development of a modern falloposcopic device that uses optical coherence tomography and wide-field imaging as complementary imaging methods to produce high-resolution images up to 1–2 mm deep into the mucosa and sub-mucosal layers of the fallopian tube [168]. The device is currently being tested in a pilot human trial for in vivo feasibility and safety [169].

Hysteroscopy is a relatively safe and routine procedure that enables clinicians to obtain images and tissue or lavage samples of areas of interest within the reproductive tract. Nonetheless, this method is quite invasive and not risk-free. Perforation of the fallopian tube or uterus is a potential risk, as well as complications from laparoscopic surgery. In addition, maneuvering these devices through small regions requires extreme skill and training, which would greatly limit widespread adoption, especially in areas where only basic care is available. Limitations such as these highlight the need for effective, accessible, and low-risk diagnostic options for patients if there is to be any positive effect on patient outcomes.

### 5.3. Emerging Imaging Techniques

A newer imaging technique, called magnetic relaxometry (MRx), detects OC through the binding of targeted iron oxide nanoparticles to ovarian cancer cells or tumor vessels. MRx uses an ultra-sensitive superconducting quantum interference detector (SQUID) to measure the magnetic field and calculate the relaxation properties of superparamagnetic iron oxide nanoparticles (SPIONs) after a brief magnetizing pulse [170]. Studies indicated that ovarian cancer lesions as small as 0.1 mm (106 cells) can be detected by labeling with SPIONs conjugated with anti-OC-associated antigens [171]. Evaluation of MRx for early detection of OC was performed by conjugating anti-CA125 antibodies with ferritin SPION nanoparticles for selectivity. Only antibody conjugated nanoparticles bound to OC cells are detected by MRx; providing exceptional sensitivity [172]. Additionally, most theories suppose OC grows from fallopian tubes; thus, MRx could in principle be applied to detect early lesions and small cancers in the fallopian tubes and ovaries.

## 6. Preoperative Diagnostics

Preoperative diagnostics are used in a setting where a woman is already suspected of having an ovarian mass, identified either through positive findings in a TVU or elevated serum CA125, and is sent for further testing perhaps due to inconclusive results. It is therefore necessary to elaborate that assays falling into this category are designed for use in women who are already scheduled for surgical follow-up and not as a standalone screening or diagnostic assay. Thus, it is important to acknowledge that the frequency of disease in the tested populations for these assays is high, driving the high positive predictive performances reported below. Detailed information for the assays discussed below can be found in Supplementary Table S1.

### 6.1. Multivariate Index Assays

**Figure 6:**
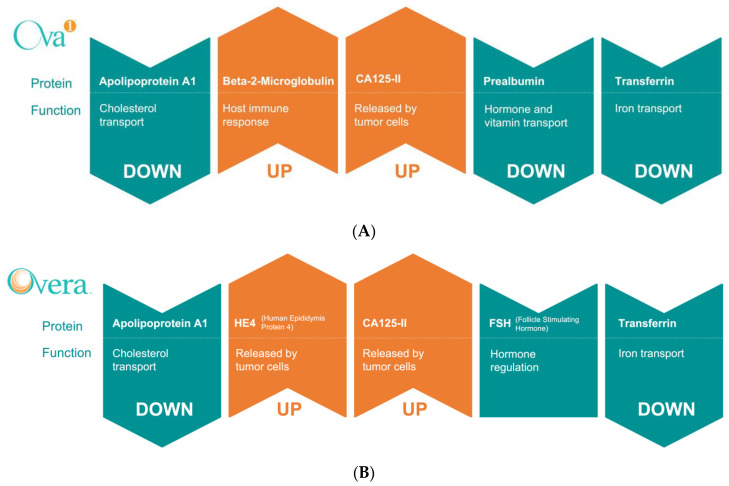
Biomarkers used in the OVA1^®^ and Overa^®^ assays developed by Aspira Women’s Health Inc. (**A**) The OVA1^®^ assay incorporates TVU findings and menopausal status with changes in serum proteins ApoA-1, B2M, CA125, Transthyretin (Prealbumin) and Transferrin. (**B**) The Overa^®^ assay is the second-generation model which incorporates two new serum biomarkers HE4 and FSH, along with three of the original biomarkers defined in the OVA1^®^ assay. Image was recreated with permission from Aspira Women’s Health [176].

There are currently two serum biomarker assays that have received clearance by the FDA for preoperative assessment of adnexal masses. In 2009, Vermillion Inc., Austin, TX, USA (now known as Aspira Women’s Health Inc., Austin, TX, USA) received clearance for its multi-marker serum assay OVA1^®^ (Figure 6A), a proprietary multivariate index assay (MIA) that is used to generate a risk score that provides an estimate of the probability of malignancy for adnexal masses. The algorithm incorporates TVU imaging and menopausal status with five serum markers, CA125, apolipoprotein A1 (ApoA-1), beta-2 microglobulin (B2M), transferrin (TF), and transthyretin (TTR), to stratify patient risk into a range of 0–10, with 10 being the highest risk of malignancy (Table 1 and Table S1) [80]. The identification and rationale for the addition of these five markers in the OVA1^®^ assay have been previously described [106,173,174]. The clinical performance of OVA1^®^ was evaluated in a number of clinical trials [80,81,83,175]. In 2011, Ueland et al., published the results of a large multi-institutional clinical trial evaluating the clinical use of OVA1^®^ in women scheduled for surgery for suspected ovarian carcinomas [80]. The authors of the study evaluated the clinical benefit of adding the OVA1^®^ test to physician assessment (PA). They found that the addition of the OVA1^®^ test greatly increased sensitivity in correctly identifying malignant cases, including epithelial and non-epithelial as well as borderline ovarian tumors stages I-IV, albeit with severely reduced specificity and PPV (PA alone: Sens. = 79%, Spec. = 75%, PPV = 62%; OVA1^®^+PA: Sens. = 96%, Spec. = 35%, PPV = 40%) [80]. A second clinical trial published in 2013, evaluating the use of OVA1^®^ for women scheduled for surgical follow-up, showed similar results (PA Alone: Sens. = 73.9%, Spec. = 92.5%, PPV = 69.4%; OVA1^®^+PA: Sens. = 95.7%, Spec. = 50.7%, PPV = 30.8%) [81]. Similar preoperative performance of the OVA1^®^ assay has been replicated in numerous peer-reviewed publications [80,81,82,83,84,175]. While the high sensitivity of OVA1^®^ in combination with physician assessment is beneficial, in that many women who do have ovarian cancer are sent for surgical follow-up, the low specificity would nominally result in a large number of women without disease being sent for unnecessary surgical evaluations.

Since its inception, the OVA1^®^ test has matured into a second-generation model called Overa^®^ (MIA2G) (Figure 6B), which received FDA clearance in 2016 [87]. Overa^®^ incorporates three of the original OVA1^®^ serum markers (CA125, ApoA-1, TF) with two additional markers (HE4 and follicle-stimulating hormone) to stratify the risk of malignancy independent of menopausal status (Table 1 and Table S1) [87]. When compared head-to-head with OVA1^®^, Overa^®^ provided a significant improvement in specificity and PPV (OVA1^®^: Spec. = 54%, PPV = 31%; Overa^®^: Spec. = 69%, PPV = 40%) [87]. Aspira has recently released a third-generation model, OVA1^®^Plus, which rather than an altogether new algorithm, is a “reflex process” model, where OVA1^®^ is performed first and if a patient falls into the intermediate categories, then Overa^®^ will be performed. While there is currently no published clinical trial or peer-reviewed data available for the OVA1^®^Plus assay, Aspira’s internal data show that OVA1^®^Plus saw a 94% improvement in the rate of cancer missed. Due to its infancy and lack of clinical trial data, OVA1^®^Plus has not received FDA clearance at this time.

### 6.2. The Ovarian Risk of Malignancy Algorithm

The second FDA-cleared protein biomarker assay for preoperative adnexal mass assessment is the Ovarian Risk of Malignancy Algorithm (ROMA^®^), developed by Fujirebio Diagnostics Inc., Tokyo, Japan, which received its clearance in 2010 [72]. Similar to OVA1^®^, the ROMA^®^ algorithm is a predictive index that combines serum measurement of two blood markers, CA125 and HE4, with menopausal status (Table 1 and Table S1) to generate a numerical risk score that stratifies women with ovarian masses into high vs. low predicted risk of malignancy. In 2011, Moore et al. published results of a clinical trial evaluating the diagnostic potential of ROMA^®^ and found that the test exhibited a high sensitivity in distinguishing ovarian cancer in TVU-positive women at moderate specificity (Sens. = 93.8%, Spec. = 74.9%) [69]. Importantly, they found that ROMA^®^ correctly identified 85% of women with early-stage epithelial ovarian cancer. A 2014 meta-analysis comparing the diagnostic accuracy of ROMA^®^ with single marker HE4 and CA125 found that ROMA^®^ aligned more with CA125 and was more suitable for diagnosing postmenopausal ovarian carcinomas, whereas the additional specificity afforded by the inclusion of HE4 made it better at identifying premenopausal malignancies [177]. These results mirrored findings from a previous meta-analysis published in 2012 reporting that HE4 had higher specificity (0.94, 95% CI = 0.90–0.96) than ROMA^®^ (0.84, 95% CI = 0.79–0.88) and CA125 alone (0.78, 95% CI = 0.73–0.83) [78]. In 2015, Grenache et al. were the first to publish a comparison of the clinical utility of ROMA^®^ with OVA1^®^ and reported that both tests performed similar with no significant difference in sensitivity, however, the ROMA^®^ test provided greater specificity than OVA^®^ (83% vs. 55%, respectively; *p* < 0.0001) [85]. In the following year, a clinical trial performed in Italy showed the ROMA^®^ algorithm was able to differentiate between benign and malignant ovarian masses with a specificity of 98%, a sensitivity of 88% and PPV of 97.8% in post-menopausal women [74]. In 2019, results of another clinical trial comparing ROMA^®^ to the second-generation Overa^®^ test demonstrated that ROMA^®^ provided statistically significant higher specificity but lower sensitivity than Overa^®^ (Spec. = 78.9% vs. 65.5%; Sens. = 79.2% vs. 91%, respectively) [86]. However, prospective studies looking at the clinical benefit of the ROMA^®^ assay for OC diagnosis show conflicting evidence, suggesting that the ROMA^®^ algorithm provides no advantage to patients over CA125 or HE4 makers alone [178,179]. Overall, while OVA1^®^, Overa^®^, and ROMA^®^ tests are currently FDA-cleared for assessing epithelial ovarian cancer individually, they are not intended to be used as a screening or stand-alone diagnostic test and should not be used without additional imaging or clinical evaluations. Sequential testing strategies that may take advantage of the high sensitivity of OVA1^®^ and the high specificity of ROMA^®^ may improve their clinical diagnostic performance.

### 6.3. The Risk of Malignancy Index

Other algorithm-based protein biomarker tests have been proposed to be used as preoperative diagnostics for OC. The Risk of Malignancy Index (RMI) was proposed by Jacobs et al. in 1990 [180]. RMI takes into account menopausal status, TVU imaging, and CA125 serum levels, and employs a simple formula of RMI = M × U × CA125, where values over a threshold score of 200 showed a strong relationship with a high risk of malignancy yielding a reported sensitivity of 96.9% and specificity of 85.4% [180]. However, further investigations into the clinical potential of the RMI algorithm were unable to replicate this diagnostic performance. In a large meta-analysis published in 2016, Meys et al. reviewed data from approximately 20,000 adnexal masses [75]. The authors of the study found that assessment of ultrasound imaging via simple rules (classifying inconclusive findings as malignant) alone performed better than RMI (93% sens. at 80% spec.; 75% sens. at 92% spec., respectively) [75]. Since its initial publication in 1990, four additional variations of the RMI algorithm have been proposed (RMI 2–RMI 5) that alter the point value changes corresponding to ultrasound and menopausal status (RMI-2 and RMI-3) [66,181], or incorporate additional variables such as lesion size (RMI-4) [67] or Doppler blood flow of the ovarian mass (RMI-5) [182]. While these versions of the assay were reported to improve sensitivity and specificity, comparative analyses have shown no significant difference in the performance of these algorithms [183]. Further modifications, such as increasing the cutoff threshold, have also been proposed to increase specificity [184]. A recent 2020 publication compared different cutoff values across all five indices and found that a cutoff of 300 performed best and that RMI 1, 2, 3, and 5 had similar positive predictive values ranging from 50–60% [185].

### 6.4. The Copenhagen Index

The Copenhagen Index (CPH-I), proposed by Karlsen et al. in 2015, provides a malignancy risk score based on the serum levels of CA125 and HE4, along with patient age (Table 1 and Table S1) [68]. In their multi-center study, the authors reported that CPH-I showed similar specificity as the established ROMA^®^ and RMI indices when sensitivity was fixed at 95% (67.3%, 70.7% and 69.5%, respectively). The preoperative performance of CPH-I in comparison to ROMA^®^ and CA125 alone was further evaluated in several independent studies around the world with similar findings [73,76,77,79,186]. The most recent validation study of the Copenhagen Index illustrated that CPH-I (AUC 0.92; 95% CI: 0.85–0.98) was able to outperform a modified ROMA^®^ index (AUC 0.54; 95% CI: 0.38–0.69), but was also comparable to a biomarker panel of six serum proteins (AUC 0.90; 95% CI: 0.82–0.97) [70]. Carreras-Dieguez et al. also assessed the preoperative performance of CPH-I and ROMA^®^ in comparison to the markers HE4 and CA125 [71]. They reported that in postmenopausal women, and women with stage I lesions, CPH-I (AUC 0.955, AUC 0.901, respectively) and ROMA^®^ (AUC 0.953, AUC 0.909, respectively) outperformed single markers HE4 (AUC 0.905, AUC 0.856, respectively) and CA125 (AUC 0.933, AUC 0.810, respectively) [71]. While the specificity of CPH-I is generally lower than the other algorithms, it can be contended that the test has considerable advantages over other methods since it is independent of menopausal status and TVU imaging, and could thus be employed in places where access to sonography is limited. In general, the data illustrate that while ROMA^®^ and CPH-I exhibit similar clinical performance, the simplicity of CPH-I may make it a better option for places where only basic health care is available and can be used to determine which patients should be referred to additional health care centers where more intensive follow-up can be given.

## 7. Genetic Diagnostics

The transformation of cells from normal to malignant state is driven by changes to cellular DNA, the core instructional code for survival, development, and reproduction. As such, cancer is commonly referred to as a genetic disease. An individual can acquire cancer-promoting alterations in the genetic code either through inherited germline passage of DNA mutations or through somatic mutations that accrue during a person’s lifetime. While most mutations in the genetic code do not significantly alter cellular phenotype, so-called “driver” mutations, such as *BRCA1/2*, are considered high penetrance mutations that confer women with a substantially higher risk of developing breast and/or ovarian cancer during their lifetime. Likewise, moderate penetrance mutations are associated with varying levels of risk that are inconsistent and often depend on family history [187]. Importantly, while the presence of driver mutations may increase risk, the frequency of those mutations in the disease population may still be low, making them poor diagnostic markers. A summarized table of the genetic-based assays presented below can be found in Supplementary Table S3.

When comparing the presence and frequency of somatic driver gene mutations in epithelial cancers, HGSC is an outlier. Whereas other epithelial cancers contain four or more driver gene mutations on average, HGSC generally only presents with one common mutation, namely *TP53*. In contrast, colorectal cancer is associated with significantly high rates of mutations in *TP53*, *KRAS*, *SMAD4*, *APC*, *FBXW7*, and *TCF7L2* [188]. Similarly, renal cell carcinomas are associated with a high frequency of gene alterations in *SETD2*, *BAP1*, *PBRM1*, and *VHL* [188]. Indeed, even other gynecological epithelial cancers such as endometrial cancer are associated with the presence of numerous driver gene mutations in *PIK3CA*, *ARID1A*, *PTEN*, *KRAS*, *CTNB1*, and *FGFR* [188]. The development of HGSC screening assays based on genetic mutations is therefore challenging since *TP53* is often the only driver gene mutated and would be quite non-specific given that *TP53* is the most commonly mutated gene across all cancer types [188] and is also known to be present in benign ovarian tissue, compromising its performance as a screening biomarker [189,190]. Furthermore, for many patients, the origins of *TP53* mutations detected in circulating DNA have been linked to the phenomenon of clonal hematopoiesis [191]. However, it would be improper to say that *TP53* is the only universal gene mutated in HGSC, and indeed, genome-wide studies such as the TCGA Program have identified a few other somatically mutated genes with a low, but still present, mutation frequency, including; *CSMD3* (6%), *FAT3* (6%), *BRCA1* (3%) and *BRCA2* (3%) [20,192]. When looking across all ovarian cancer subtypes genes involved in the PI3K–Akt pathway (*PTEN* and *PIK3CA*), genes associated with the MAPK pathway (*KRAS* and *BRAF*), and other oncogenic genes (*CTNNB1* and *PPP2R1A*) were also found to be mutated at a moderate frequency [1,192]. Genetic screening tests focused on identifying these mutations were proposed by a few groups and are discussed below.

Cell-free DNA (cfDNA) refers to extracellular fragmented nucleic acids in human biofluids [193,194]; these nucleic acids are believed to be released from cells through apoptosis, necrosis and possibly active secretion [194]. Cancer patients were found to have elevated levels of cfDNA compared to healthy individuals [193], however, cfDNA levels are also known to increase with conditions of tissue stress, such as exercise [195], trauma [196], infection [197], or transplantation [198]. Circulating tumor DNA (ctDNA) refers to cfDNA specifically derived from tumor cells and generally constitutes only a minor fraction of the total cfDNA in a given biofluid [193]. Cells, cellular debris and cfDNA found in tumor proximate fluids can also be assayed for the presence of cancer-specific genetic changes.

Recent studies have concluded that HGSC most commonly originates in the fallopian tubes, as opposed to the ovaries themselves [199]. Precursor lesions found in the fallopian tube, denoted serous tubal intraepithelial carcinomas (STICs), are characterized by the presence of mitotic figures, nuclear atypia, apoptotic bodies and loss of polarity in non-ciliated tubal epithelial cells [1,200]. STICs also present with abnormal p53 staining and an increase in Ki-67 labeling [1,200]. Even earlier lesions, known as secretory cell outgrowths (SCOUTs), were also identified as histopathologically present with a high p53 staining but lack the Ki-67 labeling seen in STICs [17]. The recent discovery of STICs and SCOUTs presents a potentially revolutionary advancement for ovarian cancer diagnostics, as screening for these lesions through minimally invasive methods would promote earlier detection of ovarian cancer and increase the number of patients diagnosed at more treatable stages.

**Figure 7:**
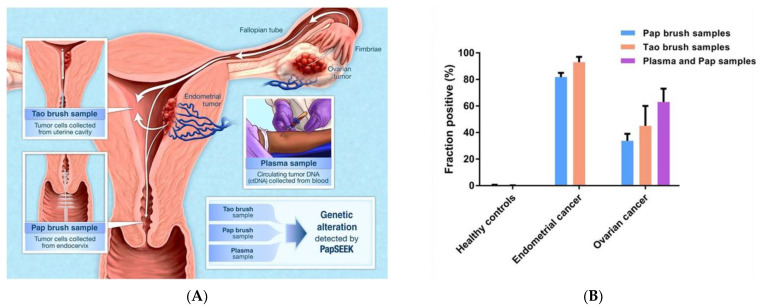
PapSEEK, proposed by Wang et al. [90], (**A**) DNA extracted from patient cytologic fluids and plasma is assayed for genetic alterations. (**B**) Target genes assayed for mutations were found in cytologic fluid for endometrial cancer samples and in both cytologic fluids and plasma for ovarian cancer samples but not in healthy controls. Used with permission.

In their seminal but limited study of 46 patients, Kinde et al. proposed investigating cells and cfDNA derived from Pap specimens for genetic alterations [192]. The resulting method, termed the “PapGene” test, employs the so-called Safe Sequencing System (Safe-SeqS) to achieve reliable detection of mutations from the cells and cellular debris collected via Pap specimens in any of 12 genes (*APC*, *AKT1*, *BRAF*, *CTNNB1*, *EGFR*, *FBXW7*, *KRAS*, *NRAS*, *PIK3CA*, *PPP2RIA*, *PTEN*, and *TP53*) found to be mutated in ovarian and endometrial cancers [192]. The authors reported that in their multiplexed assay, mutations in one or more of these 12 genes were found in 100% and 41% of endometrial and ovarian cancer cases analyzed, respectively. Importantly, none of these mutations were detected in the women without cancer that were tested [192]. In a follow-up, proof-of-principle study, the same group published PapSEEK (Figure 7), a genetic test using DNA recovered from endocervical and other biofluids. PapSEEK exhibited 33% sensitivity at 99% specificity for the detection of ovarian cancer from Pap specimens alone. The authors reported that using a Tao brush to sample fluids from inside the intrauterine cavity was able to increase sensitivity to 45% and specificity to 100%. Moreover, combining targeted sequencing for circulating tumor DNA in both patient plasma and Pap fluids further increased sensitivity to 63% at 100% specificity (Table 1 and Table S3) [90]. A caveat to reiterate however is that p53 mutations detected in uterine lavage can often be nonspecific with respect to the presence of cancer as other studies have reported that low-frequency p53 mutations are present in healthy tissues of the majority of women of all age groups [189]. The minimally invasive collection of Pap and Tao brush fluids employed by PapSEEK and PapGene, paired with the high diagnostic performance of the assays, has nonetheless highlighted the potential for Pap-based genetic testing.

In related work, Maritschnegg et al. proposed assaying uterine lavage samples as a method for identifying ovarian cancer [201,202]. They proposed that the peristaltic characteristics of the fallopian tubes allow for the transport of exfoliated cells from ovarian tumors and STIC lesions toward the uterine cavity, where they could be subsequently assayed. Consequently, they reported that *TP53* mutations were detectible in uterine lavages of 60% of ovarian cancer cases tested [201], illustrating the potential use of this approach in screening applications. This group further extended this study by employing uterine and tubal lavage along with an intrauterine catheter, which was determined to be a safe, effective, and well-tolerated method for collecting lavage samples [202].

Detection of somatic *TP53* mutations from tampons of women with HGSC was also explored. In a pilot study, Erickson et al. illustrated that DNA from exfoliated tumor cells could be assayed from tampons that had been placed in the vaginal cavity of women with ovarian cancer [203]. It was reported that tumor-derived DNA could be detected in 60% of the tampons sampled from women with intact fallopian tubes [203]. These data further suggest that exfoliated tumor cells can be assayed throughout the reproductive tract, including the vagina, and highlight promising implications for this method to be used in the non-invasive screening or detection of ovarian cancer.

Genetic-based diagnostic approaches are not limited to the detection of mutations. Copy number variations, low-frequency allele alterations, and chromosomal rearrangements are additional genetic changes that can contribute to tumorigenesis. The development of ultrasensitive techniques such as BEAMing (beads, emulsion, amplification, magnetics) [204] and droplet-based digital PCR (ddPCR) [205] have allowed for the detection of mutations, copy number changes, and low-frequency alleles alterations down to 0.01% in cfDNA. Next-generation sequencing (NGS) data from whole-genome sequencing (WGS), whole-exome sequencing (WES), and targeted sequencing (e.g., molecular-barcoding, amplicon-based and hybrid-based approaches) were shown to enable the detection of higher coverage regions for detecting chromosomal aberrations with improved detection limits [193,194]. WES allows for the identification of alterations in protein-coding regions across the genome, and WGS enables analysis of non-coding regions and has a high sensitivity for copy number variants and structural rearrangements [206]. The clinical use for WES and WGS, however, has remained limited due to their high cost, difficulties in data interpretation, and the high number of variants of uncertain significance [206,207]. Despite these limitations, WGS and WES evaluation of women presenting with ovarian masses has exhibited similar diagnostic potential as preoperative assays based on traditional serum-based protein markers (covered in more detail in Section 6). In a comparison study, Chen et al, assessed the performance of a risk of malignancy (RM) calculated from copy number variations, as determined by low-coverage WGS, against cancer markers CA125, HE4, and the ROMA^®^ index. They reported that the diagnostic potential of RM (AUC 0.837) was similar to the traditional ROMA^®^ (AUC 0.876) and HE4 (AUC 0.866) assays, and performed better than CA125 (AUC 0.775) [208].

Overall, the rapid progression of sequencing technologies and the recent development of minimally invasive cfDNA-based platforms for genetic mutation screening have changed the outlook for genetic-based diagnostic approaches. With the introduction of massively parallel sequencing, researchers and clinicians can now interrogate cancer at the genome-wide scale, and liquid biopsies, either from a blood draw or other fluid collection, offer minimal risk to patients and appear to be a promising alternative to solid tumor biopsies.

## 8. Epigenetic Diagnostics

Epigenetics describes the study of heritable cellular processes that alter gene expression or chromatin state without altering the genetic sequence itself [209]. While various mechanisms of gene regulation fulfill these criteria, including DNA methylation, histone modifications and noncoding RNA, it is DNA methylation that remains the most widely studied. DNA methylation occurs via the addition of methyl (CH_3_) moieties to cytosine residues of 5′-cytosine-phosphate-guanine-3′ (CpG) dinucleotides, of which there are roughly 30 million instances throughout the human genome. These relatively minor alterations in chemical structure can nonetheless have profound effects on gene expression, particularly in the context of dense clusters of CpG dinucleotides known as “CpG islands” (CGIs) often located in gene promoter regions, where they can dramatically alter cellular phenotype through methylation-mediated gene silencing. Numerous studies have now demonstrated that virtually all human cancers exhibit widespread alterations in DNA methylation that can occur at the earliest stages of cancer and can contribute to carcinogenesis through the silencing of key tumor suppressor genes. Biomarkers based on cancer-specific DNA methylation are an attractive option for cancer diagnostics, as these alterations typically occur in hundreds to thousands of different regions throughout the cancer genome that can be used to create panels of methylation biomarkers to improve achieve higher levels of clinical sensitivity and specificity. In addition, DNA methylation patterns are highly specific to the cell(s) of origin, which various studies have demonstrated can be leveraged to successfully identify cancer tissues of origin, including discrimination of etiologically diverse ovarian carcinomas. A summarized table of the epigenetic-based assays presented below can be found in Supplementary Table S3.

The first wave of research studies investigating cancer-associated DNA methylation was primarily limited to exploring the prevalence of methylation in the promoter regions of classical tumor suppressor genes in various cancer types. In the context of ovarian cancer, such tumor suppressor genes included *BRCA1*, *RASSF1A*, *E-cadherin* (*CDH1*), *HIC1*, *APC* and the *HOX* genes [210,211], among others. A particularly interesting finding was the identification of a high prevalence of hypermethylation in the promoter region of the Opioid Binding Protein/Cell Adhesion Molecule-Like (*OPCML*) gene in women with HGSC. *OPCML* was discovered through the investigation of the loss of heterozygosity at the 11q25 locus associated with HGSC and was subsequently confirmed to function as a tumor suppressor that continues to be explored as a potentially useful biomarker of ovarian cancer (Table 1 and Table S3) [91,92,212]. During this time, other seminal studies began to establish that cancer-specific alterations in DNA methylation could often be detected in circulating DNA in the blood of cancer patients, including women with ovarian cancer [213,214,215]. Additionally, during this time, and in contrast to other studies focused on DNA hypermethylation, Dammann et al. reported a high prevalence of DNA hypomethylation in HGSC tumors [216] at several loci, most notably LINE-1 retrotransposons, which were later confirmed to become demethylated during precursor stages of ovarian carcinogenesis [217].

The introduction of NGS and DNA microarray technologies circa 2010 ushered in a new horizon for DNA methylation-based diagnostics. Whereas early epigenetic studies were largely limited to investigating locus-specific methylation of previously identified tumor suppressor genes in a serial manner, these technologies afforded researchers the new ability to explore methylation alterations occurring throughout the entire genome. The result was a plethora of studies aimed at profiling genome-wide methylation (the “methylome”) of numerous cancer types, including ovarian cancer. The main conclusion of these studies was that cancer-associated aberrant methylation is not limited to promoters and CGIs, but can also be found in thousands to tens of thousands of loci in numerous genomic contexts throughout the genome. This revelation greatly facilitated the discovery of a multitude of candidate cancer-specific methylation biomarkers that could be selected and combined into panels to achieve higher levels of clinical sensitivity and specificity for the detection of various cancer types, including ovarian cancers.

The first large-scale study of methylomic analyses of ovarian cancers was accomplished in coordination with The Cancer Genome Atlas (TCGA) consortium, which employed Illumina’s first-generation HumanMethylation BeadChip 27, containing probes for 27, 578 CpG sites throughout the genome [20]. The data from these and other early BeadChip 27 analyses were made publicly available for bioinformatic analyses that paved the way for the identification of novel methylation biomarkers that could be tested and validated in numerous subsequent studies [212,218,219,220,221]. There are, however, some notable limitations to these initial ovarian cancer methylation discovery datasets, namely the relatively low genomic coverage (<0.1% of all CpG sites) and inclusion of only a small number of adjacent normal control samples derived from patient fallopian tubes, which is now widely accepted as the tissue-of-origin for the majority of HGSCs. A pair of subsequent, independent studies by Sánchez-Vega et al. [94] and Widschwendter et al. [93], respectively, addressed some of these limitations by leveraging Illumina’s second generation, Infinium 450K BeadChip to profile HGSC tumors and fallopian tube controls, leading to the identification of novel methylation biomarker panels. Interestingly, these studies independently identified *ZNF154* methylation as a key biomarker of HGSC and were able to use its methylation status to achieve relatively high levels of diagnostic performance in blood of 48.5% sensitivity at 93.5% specificity [93] and 86.8% sensitivity at 93.5% specificity [95] (Table 1 and Table S3). Indeed, a recent meta-analysis of studies investigating liquid-biopsy-based detection of OC concluded that methods based on analysis of DNA methylation, particularly since the advent of methylome BeadChip arrays, generally offer superior performance over other molecular diagnostic strategies [222]. In another interesting study by Bodelon et al., the authors performed a 450K BeadChip analysis of the major pathologic subtypes of ovarian cancers and found that ovarian tumors can be readily classified into one of four OC methylation subgroups, which were hypothesized to reflect the etiology of each respective tumor and which lead to distinct differences in survival [223] (Supplementary Table S3).

As previously mentioned, current research now indicates that most HGSCs actually originate as serous tubal intraepithelial carcinomas (STICs) of the fallopian tubes and that the ovary is involved secondarily and only at late stages [16,224,225]. It has thus been argued that a true reduction in the burden of ovarian cancer will ultimately require a means of detecting early-stage tumors and even precursor STIC lesions. Toward this end, in a pair of independent studies, we expanded upon prior methylomic studies by performing genome-wide methylation analyses of HGSC tumors, as well as fallopian tube precursor lesions, using the third generation, Illumina MethylationEPIC array, which enabled characterization of over 850,000 or ~3% of all CpG sites throughout the genome [200,226]. The increase in genomic coverage afforded by the Methylation EPIC platform ultimately enabled the identification of a set of 91 and 42 loci exhibiting HGSC- and STIC- specific hypermethylation, respectively, when compared to healthy gynecological tissues. Importantly, there was considerable overlap (>40%) in the methylation biomarkers in these studies, with several achieving high levels of clinical sensitivity and specificity indicating that epigenetic alterations arise at very early stages of ovarian carcinogenesis. This comports well with a previously published report demonstrating the occurrence of epigenetic reprogramming in the fallopian tube fimbriae of *BRCA* mutation carriers [227]. Taken together, the results of these studies indicate that methylation biomarkers detection could hold promise for the detection of HGSC at early, and even precursor, stages of disease [228].

## 9. Pan-Cancer Screening

Pan-cancer screening describes diagnostic techniques aimed at detecting and identifying multiple cancer types. Pan-cancer approaches are typically based on the detection of tumor-specific changes in circulating tumor DNA, proteins, metabolites, etc. for upwards of 100 different cancer types from a single blood draw. These methods also commonly employ machine learning algorithms paired with multi-analytic assay strategies to achieve pan-cancer detection. In principle, pan-cancer tests hold the potential to bring a revolutionary change to cancer screening, in that only a single blood draw would be necessary for early-stage detection of multiple cancer types. However, it is important to note that multi-cancer screening tests in the general population would need to be designed and implemented in such a way to carefully balance specificity and sensitivity for each tumor type and a reliable means of identifying tissues of origin. It has therefore been posited that the most impactful benefit of pan-cancer screening would not be to diagnose disease, but rather to identify individuals that may need more extensive diagnostic follow-up. A summarized table of the pan-cancer assays presented below can be found in Supplementary Table S3.

### 9.1. FDA Approved Pan-Cancer Screening

As of 2020, the FDA has approved only one pan-cancer screening assay that includes testing for ovarian cancer. The FoundationOne^®^ Liquid CDx (F1LCDx) was developed by Roche Holding AG, Basel, Switzerland (PMA P200006) [229] and is intended for use as a companion diagnostic to identify patients that would benefit from targeted cancer therapies. The NGS-based test employs hybridization capture of cfDNA isolated from plasma to identify specific DNA mutations, copy number alterations, gene rearrangements, microsatellite instabilities, and tumor mutational burden. Results of F1LCDx are used to inform clinicians of which targeted therapy approaches would benefit patients given their clinical indication. As an example, detection of *BRCA1/2* alterations in an ovarian cancer patient might guide clinicians to implement a PARP inhibitor chemotherapeutic strategy [229].

### 9.2. Pan-Cancer Tests in Development

Galleri^®^, developed by GRAIL, Menlo Park, CA, USA was launched as a ‘Laboratory Developed Test’ in 2014 and, although it is currently not approved or cleared by the FDA, is still able to be marketed and sold under a CLIA waiver to selected consumers. The Galleri^®^ test is purported to detect over 50 different cancer types, including ovarian cancer, via NGS-based analysis of DNA methylation signatures of cfDNA derived from a single blood draw. The Galleri^®^ assay exhibited an impressive 99.3% specificity for both early and late-stage cancer detection across all cancer types tested, however, for early-stage tumors specificity decreased to 43.9% in all cancer types [230]. Assay sensitivity metrics followed similar results with sensitivity increasing with increasing stages, 18% in stage I to 93% in stage IV, across all cancer types [230]. The ability to detect tissue of origin with the Galleri^®^ test was remarkably high across all cancer types, with a 93% accuracy. Galleri^®^ is currently not yet commercially available to the general public, however, those who are enrolled in GRAIL’s clinical trials are able to purchase the test out of pocket [231].

CancerSEEK is a pan-cancer test originally reported in 2018 by Cohen et al. [96], and later launched by Thrive Earlier Detection Corp, Cambridge, MA, USA. The test uses a combination of 39 protein markers and amplicon-based cfDNA sequencing to detect a total of 16 major types of cancer, many of which currently do not have screening options available to patients. When looking specifically at its performance for ovarian cancer, CancerSEEK achieved a remarkable sensitivity of 98% at 99% specificity (Table 1 and Table S3). It has received the FDA’s Breakthrough Device designation for detection of genetic mutations and was bought by Exact Sciences Corp., Madison, WI, USA in 2020. While early data from CancerSEEK appear to be very promising, its performance has yet to be independently validated and the assay is still in development and not currently available to patients.

Targeted error correction sequencing (TEC-Seq), reported by Phallen et al. used machine learning and NGS-based high throughput sequencing to identify a mutational profile of ctDNA isolated from plasma [232]. Colorectal cancer and ovarian cancer exhibited the highest sensitivity for the TEC-seq assay across all disease stages (I-IV), achieving 83% and 71% sensitivities for these cancer types, respectively. When looking only at early-stage disease, TEC-seq was 67% and 75% sensitive in identifying ovarian cancer at stages I and II, respectively. Importantly, TEC-seq did not detect mutations in any of the healthy controls assayed, giving the test a very high specificity of >99.99% [232].

**Figure 8:**
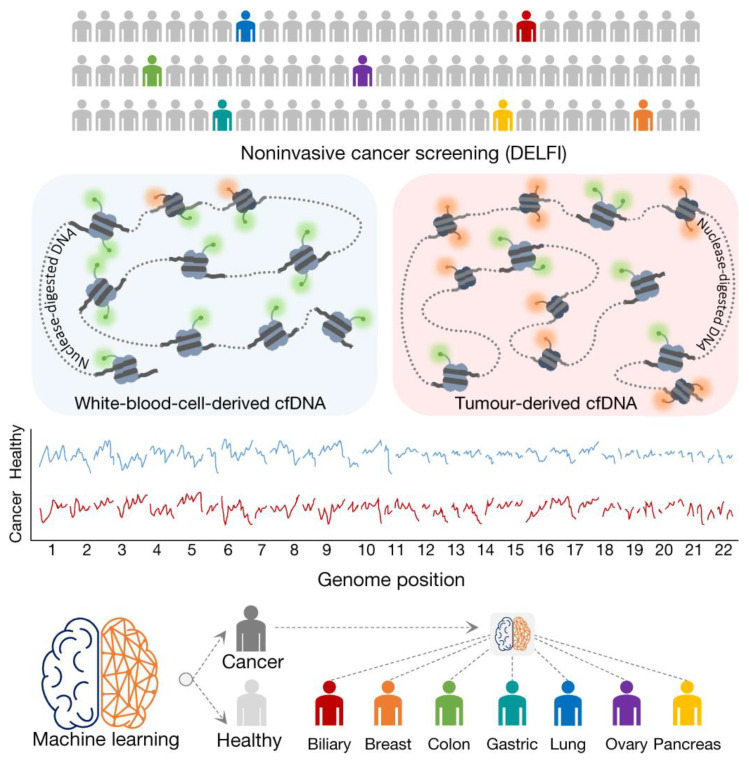
Schematic of DELFI approach. Cristiano et al. [233]. Cancer-specific fragment profiles are generated through WGS of cfDNA isolated from plasma and compared to profiles generated from healthy individuals. Unique fragment alterations at specific loci can be mapped back to detect and identify specific cancer types. Used with permission.

Lastly, Cristiano et al. proposed a new screening approach termed DELFI, or “DNA evaluation of fragments for early interception” (Figure 8) [233]. DELFI uses machine learning paired with low coverage WGS to study variations in cfDNA fragmentation patterns between healthy individuals and those with cancer. DELFI’s fragmentation profiles exhibited an AUC of 0.99 at 95% specificity for ovarian cancer patients [233].

The evaluation of cfDNA/ctDNA for multi-cancer screening panels is clearly an area of active research as both pharmaceutical and academic institutions are working in tandem to identify accurate and reliable assays for early detection. Recent advancements in sequencing and machine learning strategies have greatly propelled such research efforts forward, and while the majority of pan-cancer technologies are still in the developmental pipeline, results from large clinical trials are greatly anticipated as positive results would surely be a major breakthrough for reducing cancer mortality and morbidity worldwide.

## 10. Emerging Diagnostic Approaches

Ovarian cancer diagnostic methods are currently constrained to a pelvic exam, imaging and the measurement of serum protein CA125 [234]. While these approaches are the mainstay of ovarian cancer diagnostics, 70% of HGSC cancer patients still fail to be diagnosed until late stages [5,7], and there thus remains a critical need to identify better diagnostic biomarkers for early detection. The addition of newly identified biomarkers discussed below are currently emerging to complement the existing clinical practice with an aim to increase early detection, as some of these markers may show changes well before an increase in serum CA125 can be observed. A summary of the diagnostic and screening tests presented below can be found in Supplementary Table S4.

### 10.1. MicroRNA

MicroRNAs, or miRNAs, are a family of noncoding RNAs that play critical roles in regulating gene expression. Typically only 20–25 nucleotides in length, miRNAs influence the expression of particular target genes by interacting with mRNAs, leading to mRNA degradation and translational suppression. It is now well established that miRNAs often become highly dysregulated during carcinogenesis leading to significant changes in cell phenotype that can contribute to the initiation and progression of human malignancies, including ovarian cancers [235,236]. Furthermore, miRNA expression is highly tissue-specific leading to specific expression profiles that can potentially be leveraged to improve cancer detection, as well as to identify tissues of origin.

While a detailed survey of miRNA-based OC diagnostics literature is beyond the current scope, we highlight here a few of the most important and promising studies and refer the reader to any of the excellent comprehensive reviews that have recently been published on the subject [235,237]. The first major study of miRNA dysregulation in ovarian cancer was reported by Iorio et al. in 2007 [97]. The authors found that numerous miRNAs were recurrently upregulated (e.g., miR-200a, miR-141, miR-200c and miR-200b) or downregulated (e.g., miR-199a, miR-140, miR-145 and miR-125b1) in ovarian tumors and that the corresponding miRNA expression levels could be used to reliably identify and classify ovarian tumors from healthy ovarian tissues (the fallopian tube origin hypothesis was only just emerging at the time) according to their histologic subtype [97]. Subsequently, published investigations have confirmed and extended these results, leading to the identification of a host of dysregulated miRNAs, including many demonstrating potential utilities as biomarkers for early diagnosis, prognosis and monitoring of ovarian cancer. At the same time, a number of other major studies demonstrated that miRNAs are highly stable biomolecules that can be reliably detected in cell-free form in numerous biofluids, including blood, urine and saliva, to name a few [238,239,240]. These findings in particular opened the door to a wealth of investigations exploring the potential utility of miRNA profiling for noninvasive detection of cancer, including ovarian cancer.

The first prima facie evidence of the potential for noninvasive miRNA-based OC diagnostics was reported in 2008 by Taylor et al. [241]. In this seminal study, the authors demonstrated that microarray analysis could be used to identify OC-specific miRNA signatures using miRNA obtained from circulating extracellular vesicles (discussed in detail in Section 10.2) in the sera of OC patients. The authors further found that exosomal ratios of eight miRNA biomarkers (miR-21, miR-141, miR-200a, miR-200b, miR-200b, miR-203, miR-205 and miR-214) correlated with those of the primary tumor and could also be used to readily distinguish the patients with ovarian cancer from those with benign or no disease. The results of this study, combined with the discovery of the existence of free-floating circulating miRNAs, spurred a number of follow-up studies further exploring the biology and diagnostic potential of miRNAs in relation to OC. The preponderance of these studies, a summary of which can be found elsewhere [237], employed reverse transcriptase-quantitative PCR (RT-PCR), or less often RNA sequencing, to identify and quantify a wide range of previously identified and novel, dysregulated miRNAs.

More recently, several promising new approaches that leverage more advanced bioinformatic methods have been developed to improve performance for blood-based miRNA OC diagnostics. For example, Elias et al. used neural network machine learning of small RNA sequencing data from 179 serum samples to develop an algorithm for miRNA-based detection of OC [98]. The algorithm yielded 14 candidate miRNA biomarkers, which were then technically validated by RT-qPCR, leading to the selection of a final panel of seven miRNAs (miR-29a-3p, miR-92a-3p, miR-200c-3p, miR-320c, miR-335–5 p, miR-450b-5p, and miR-1307–5 p). Validation of this panel in an independent analysis of 51 specimens yielded a PPV of 91.3% (95% CI: 73.3–97.6%) and NPV of 78.6% (95% CI: 64.2–88.2%) based on the composition of the study cohort. Following a similar approach, Kandimalla et al. performed small RNA sequencing in a cohort of 31 tumor samples from women with early-stage HGSC, identifying a panel of eight miRNA biomarkers (miR-182, miR-183, miR-202, miR-205, miR-508, miR-509-3, miR-513b and miR-513c) that exhibited upregulated expression in the blood of women with OC [99]. The authors then validated this panel with RNA-seq data derived from TCGA and then evaluated the potential diagnostic utility of the panel by employing multiple logistic regression to train a diagnostic model based on an independent RNA-seq dataset from a cohort of 640 serum samples. This model was then independently validated in three retrospective and one prospective RNA-seq dataset, achieving AUC values ranging from 0.82 to 0.92. Lastly, Yokoi et al. used miRNA microarray analysis with a cohort of 1539 serum samples to develop a diagnostic model based on the expression levels of 10 miRNAs (miR-320a, miR-665, miR-3184-5p, miR-6717-5p, miR-4459, miR-6076, miR-3195, miR-1275, miR-3185, and miR-4640-5p) [101]. This model was then validated in an independent cohort of 1560 samples and achieved an astounding sensitivity of 99% at 100% specificity for the detection of ovarian carcinomas. While their initial diagnostic model was able to successfully discriminate non-epithelial ovarian cancer patients from non-cancer controls, it could not distinguish OC patients from those with borderline or benign tumors. To address this issue, the authors developed a more specific, alternative diagnostic model that was able to distinguish OC patients from women with benign tumors that yielded more modest AUC values ranging from 0.72–0.82.

Lastly, it is worth observing that the diagnostic models developed through comprehensive and elaborate bioinformatic analyses of independent miRNA datasets often lead to the identification of panels with nonoverlapping sets of miRNA biomarkers. This may reflect some of the challenges facing the field, not the least of which includes a current limited understanding of potentially complicated relationships with respect to miRNA species and human disease. Furthermore, there remain concerns that miRNA diagnostics suffers from a lack of standardization in profiling methods, which are notoriously sensitive to methodological differences, most notably the particular extraction and analysis methods employed [242,243,244,245]. These issues represent significant but likely surmountable hurdles that will need to be overcome to achieve sufficiently robust performance to enable translation into the clinic.

### 10.2. Extracellular Vesicles

Extracellular vesicles (EV) are small lipid-bound vesicles that are secreted into the intercellular space and contain small intracellular molecules such as messenger RNA (mRNA), microRNA (miRNA), small interfering RNA (siRNA), single-stranded DNA (ssDNA), double-stranded DNA (dsDNA), amplified oncogene sequences, and proteins [246,247]. EVs can be broadly classified into two groups based on their biogenesis: the first of these is known as microvesicles, ectosomes, or microparticles, and is formed by outward budding of the plasma membrane at the cell surface, incorporating surface-associated intracellular components and membrane-bound proteins in the resulting vesicles [248]. The second type of EVs develop in the late endosomal compartment through inward budding of multivesicular body membranes that engulf cytosolic components in intraluminal vesicles known as exosomes. These exosomes are released from the cell via endosomal fusion with the cell membrane [248]. Despite a clear distinction in the biogenesis of extracellular vesicles, many studies discussing the role of EVs in cancer use the generic term “exosomes” to designate the EVs without necessarily demonstrating their intracellular origin. We thus opted to use the generic term EVs or exosomes interchangeably in this review [249].

Exosomes have gained interest from researchers because the exosomal contents presumably reflect the molecular composition of their cell of origin and also contribute to intercellular communications [250]. For example, researchers found that EVs promote tumor growth, increase invasiveness, and mediate metastasis in numerous forms of cancer [249]. Several ovarian cancer studies have shown that EVs and their cargoes, such as proteins and miRNAs, can contribute to tumor progression [251] and facilitate chemotherapy resistance [252].

**Figure 9:**
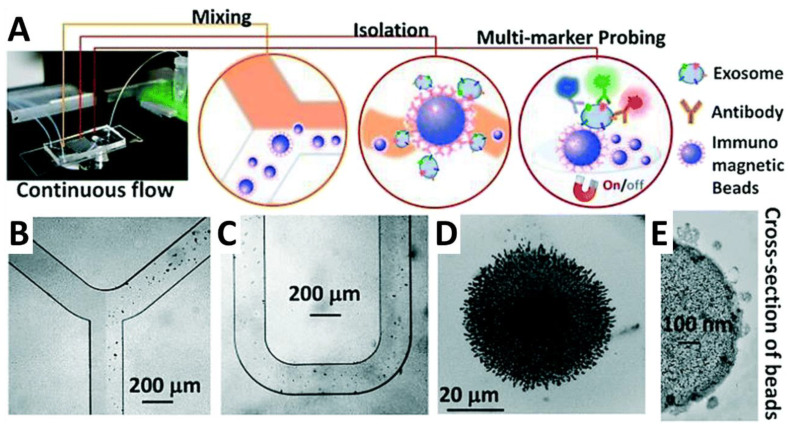
Workflow of the ExoSearch chip developed by Zhao et al. [257]. (**A**) Patient plasma (orange) is mixed with immunomagnetic beads that bind exosomes within the sample. Beads carrying exosomes are then isolated via a magnetized field in which the number of beads isolated was in direct comparison to the sample input and could be quantified. A mixture of fluorescently labeled antibodies is then applied to the isolated beads for multi-color fluorescence imaging. (**B**,**C**) Bright-field images of the immunomagnetic beads in the microfluidic compartments. (**D**) Aggregated exosome-bound immunomagnetic beads after magnetic separation (**E**) Transmission electron micrograph depicting the cross-section of an exosome-bound immunomagnetic bead. Reprinted under the Creative Commons CC BY-NC3.0 license.

Exosomal proteins have been widely explored as potential OC biomarkers. For example, in a study by Li et al., the authors looked at the presence of exosomal claudin-4, a tight-junction protein, and found that EVs in 32 of 63 plasma samples from ovarian cancer patients were positive for this protein, while only 1 of 50 samples from the healthy control group exhibited claudin-4 positive exosomes. However, at a fixed 98% specificity, the presence of exosomal claudin-4 showed decreased sensitivity (51%) when compared to serum CA125 (71%) [253]. In another study, Liang et al. discovered that overexpressed proteins in OC were also present in exosomes, including epithelial cell adhesion molecule (EpCAM), proliferation cell nuclear antigen, tubulin beta-3 chain, EGFR, apolipoprotein E (APOE), claudin 3, fatty acid synthase, ERBB2, and L1CAM (CD171), suggesting these proteins might be considered diagnostic biomarkers [254]. In another study, Runz et al. showed that the exosomal proteins CD24 and EpCAM were enriched in malignant OC ascites [255]. A recent study revealed exosomes containing soluble E-cadherin were highly expressed in ascites of OC, discriminating OC from benign conditions [256]. Zhao et al. developed the “ExoSearch chip” (Figure 9) as a means of detecting three exosomal proteins (CA125, EpCAM, and CD24) in the serum of OC patients [257]. In a pilot study of 20 human subjects, the authors reported impressive diagnostic power (AUC 1.0, *p*-value = 0.001) for their assay. The ExoSearch chip requires only 20 μL of serum and provides the results in as little as 40 min [257]. In another interesting finding, EVs were found to contain microbial DNA from *Acinetobacter*, which could be quantified and, when combined with age and serum CA125 levels, showed a diagnostic performance with 82.1% sensitivity at 68.0% specificity [258].

### 10.3. Autoantibodies

The sensitivity of tumor biomarkers for early detection is often limited by the volume of tumors as well as the expression level and shedding rate of biomarkers within the tumor environment. Due to the small size of early-stage ovarian carcinomas, biomarkers shed from tumor cells may be insufficient to raise serum levels but may nonetheless evoke patient immune responses [259]. Tumor-associated autoantibodies (AAb) are well-established biomarkers in many solid tumors and are often detected prior to clinical manifestations of disease including ovarian cancer [260].

*TP53* tumor-suppressor gene mutations are present in virtually all HGSCs and often lead to the accumulation of p53 protein in the cytoplasm [261]. Autoantibodies reactive to wild-type *TP53* were reported to occur in the sera of 15% of OC patients [262]. Elevation of p53-AAb was detected in 16% of patients whose cancers were missed by the UKCTOCS risk of ovarian cancer algorithm (ROCA). p53-AAb titer rose 11 months before CA125 was elevated and 22.9 months prior to cancer diagnosis in patients whose CA125 was not elevated. The elevation of p53-AAb provided clinically significant lead time compared to the elevation of CA125 or ROCA value, potentially enabling earlier detection of invasive OC [262].

A variety of other AAbs were also investigated. A study by Yoneyama et al. reported that RhoGDI-AAb (Rho GDP-dissociation inhibitor) detected OC with a sensitivity of 89.5% at a specificity of 80% [263]. TUBA1C-AAb (Tubulin alpha-1C chain) was reported to have a sensitivity of 89% at 75% specificity for discrimination of women with OC from those with benign conditions [264]. Another study found that expression of the homeobox gene *HOXA7* is associated with aberrant Müllerian-like differentiation in epithelial ovarian tumors and correlated with the generation of HOXA7-AAb in patients. Detection of HOXA7-AAb provided a 66.7% sensitivity with 100% specificity for moderately differentiated ovarian tumors [265].

To improve accuracy, combinations of multiple AAbs have also been explored. A panel of 11 AAb markers against a variety of proteins (ICAM3, CTAG2, p53, STYXL1, PVR, POMC, NUDT11, TRIM39, UHMK1, KSR1, and NXF3) was developed for detection of HGSC exhibiting a combined 45% sensitivity at 98% specificity [266]. A combination test including EpCAM-AAb with CA125 in serum samples identified EOC with 90.4% sensitivity at 92.3% specificity [267]. For early-stage (I-II) OC, combination measurement of serum IL-8 cytokine, IL8-AAb, and CA125 provided 87.5% sensitivity at 98% specificity [268]. Another recent pilot study for Stage I HGSC reported HSF1-AAb (Heat shock factor-1) performed best with a sensitivity of 95% at a specificity of 80% (AUC 0.95) and, when combined with CCDC155-AAb (Coiled-coil domain containing protein-155) and CA125, the assay reached a higher sensitivity of 98% with a specificity of 60% (AUC 0.94) [260].

### 10.4. Microbiome

Recent studies have begun to increasingly reveal that microbiome dysbiosis, or oncobiosis, can often play a significant role in oncogenesis. For example, metabolites from microbial taxa were shown to influence basic cellular functions such as the homeostasis of reduction-oxidation [269] and cellular metabolism [270], by altering the gene expression of human cells. These alterations can further precipitate events related to cancer development, such as angiogenesis [271], epithelial–mesenchymal transition, tumor proliferation and invasion, immune cell signaling [272], and tumor-promoting inflammation [273]. Data suggest that microbiome dysbiosis drives inflammation and immune responses to promote OC carcinogenesis, which is supported anecdotally by the fact that pelvic inflammatory disease (PID) serves as a risk factor for OC [274]. Infection of the genital tract is a strong driver of local inflammation, which in turn triggers oncogenesis through multiple pathways, including increased oxidative stress, DNA damage, and subsequent accumulation of genetic mutations [275].

Some microbiome dysbioses and metabolites were shown to be of diagnostic value for OC. Zhou et al. found the microbial composition was altered in the distal fallopian tubes of OC patients [276]. In particular, the ratio of phyla Proteobacteria to Firmicutes was notably increased in ovarian cancer due to an elevated abundance of Proteobacteria [276]. Indeed, the authors found that the species *Acinetobacter lwoffi* (AUC 0.608; *p*-value < 0.05) was increased while *Lactococcus piscium* (AUC 0.808; *p*-value < 0.05) was decreased, indicating a microbial composition change might be associated with initiation and progression of OC [276]. Recently, a group of researchers re-examined whole-genome and whole-transcriptome sequencing from the TCGA (The Cancer Genome Atlas) compendium and found that bacterial signatures in blood were predictive for early-stage (Ia-IIc) cancer patients that lacked genomic alterations. Specifically, bacterial counts identified with a stochastic gradient-boosting machine learning model for ovarian cancer patients illustrated an impressive AUC of 0.9956 and an area under the precision-recall curve (AUPR) of 0.9663 when compared to healthy tissues [277]. Furthermore, Miao et al. reported 19 microbial profiles in peritoneal fluid highly specific to OC [278]. Their analysis predicted that the inclusion of microbial profiles with serum tumor markers (CA125 and HE4) and controlled features (patient age and BMI) would increase the diagnostic accuracy (AUC 0.904) compared to current screening methods with just serum CA125 and HE4 (AUC 0.804) [278].

There has been a recent boom in oncology research focused on understanding the roles that bacteria and microorganisms that inhabit the cancer tumor microenvironment play in oncogenesis. The cancer microbiome comes with numerous challenges including inter and intra-personal variations based on an array of factors including lifestyle and genetics. Additionally, the immense complexity of the human microbiome may limit a one-size-fits-all approach. Despite the current limitations, as more data and research are published describing influential microbial populations, metabolites and small molecules, researchers may be able to leverage these new kinds of biomarkers for improved early detection and ovarian cancer diagnostics.

### 10.5. Metabolomics

Metabolomics is an emerging member of “omics” based research which looks at small molecules present in an environmental system to understand the effect of their interactions within biological networks. Biofluids of interest in metabolomics include serum/plasma, urine, cyst fluid, and ascitic fluid. Metabolites related to cellular respiration, carbohydrate, lipid, protein, and nucleotide metabolism have all been reportedly altered in patients with OC compared to healthy controls [279].

Metabolite panels derived from patient sera [102,103,104,280,281,282,283] and urine [105,284] were proposed for use in the diagnosis of ovarian cancer. In 2004, Odunsi et al. were one of the first groups to publish the use of ^1^H-NMR spectrometry for identifying ovarian cancer metabolites in patient serum [280]. The authors reported that metabolites such as alanine, leucine, 3-hydroxybutyrate, low and very low-density lipids, glutamine, cholesterol, and lactate all showed differences between healthy controls and ovarian cancer cases. The ^1^H-NMR descriptor at regions 2.77 and 2.04ppm exhibited an AUC of 1, perfectly differentiating between all cases and controls, including early-stage disease [280]. Garcia et al. also used ^1^H-NMR spectrometry to identify a similar panel of lipids, including valine, 3-hydroxybutyrate, creatine and acetone that collectively exhibited 68% sensitivity at 95% specificity for identifying ovarian cancer cases versus controls [102]. 3-hydroxybutyric acids, 3,4-dihydroxybutyric acids combined with serum CA125 exhibited an AUC of 0.98 for separating OC from healthy controls in a panel reported by Hilvo et al. [283]. Lysophosphatidylcholines (LysoPC) have emerged as important metabolites altered in ovarian cancer. Zhang, et al. showed a panel consisting of L-tryptophan, LysoPC (18:3), LysoPC (14:0), 2-Piperidinone, and two unknowns isolated from urine were able to distinguish cancer cases from controls with an AUC of 0.86 [105]. Chen et al. illustrated that a panel consisting of serum metabolites hypoxanthine, guanidinosuccinic acid, cortisol, LysoPE (22:6), LysoPE (22:6) fragment, and LysoPC (18:2) among a large list of others demonstrated 93.2% sensitivity at 92.7% specificity for identifying ovarian cancer [103].

Recently, Slupsky et al. reported that a panel of urine-derived acetone, allantoin, carnite, methanol, urea, 1-Methylnicotinamide, Levoglucosan and two unknown singlets at 3.79 and 2.82 ppm were able to distinguish ovarian cancer with 98% sensitivity at 99% specificity [284]. Large panels of metabolites associated with histamine metabolism, amino acid metabolism, phospholipid metabolism and fatty acid oxidation, among others, showed good diagnostic potential, as reported by Ke et al. (AUC 0.91) [285] and Zhou et al. (100% sens. at 98% spec.) [282]. Lastly, panels of miscellaneous metabolites described by Buas et al. [286], Fan et al. [104], and Guan et al. [281] have illustrated sensitivities of 95%, 92.1%, and 83.8% at specificities 43%, 88.6%, and 77.1%, respectively, in discriminating ovarian cancer from healthy controls.

Similar to the cancer microbiome, cancer metabolomics is a newly emerging field that remains largely unexplored for cancer screening and diagnostics. The clinical application of metabolomic approaches requires a deeper understanding of how these molecules influence physiology, cell function, and biology. Early work into the metabolites of cancer has identified key metabolic pathways that can be targeted for further evaluation by researchers. Further enhancement in detection systems and downstream analysis pipelines will propel the field of metabolomics to become a more widely used tool for cancer diagnostics and screening.

## 11. Conclusions and Outlook

Ovarian cancer is a highly aggressive gynecologic malignancy affecting women in the developed world and accounts for the death of over 12,000 women in the U.S. each year. For the majority of these women, ovarian cancer is diagnosed at a late stage leaving them with a poor chance of survival. There thus remains a tremendous need for more effective diagnostic strategies as CA125 and TVU remain the only commonly employed diagnostic methods for detecting and monitoring OC, despite a failure of these techniques to provide statistically significant benefit in terms of patient mortality. Nonetheless, there remains tremendous reason for hope. A new, more accurate understanding of the etiologies of this heterogenous group of diseases is leading to the discovery of a host of novel biomarkers. The development of noninvasive diagnostic approaches, including liquid biopsy, Pap specimen and pan-cancer techniques, is being driven by increasingly powerful machine learning algorithms while emerging technologies such as microchip and nano-based platforms are only just now touching the surface. A variety of new cancer biomarker types, such as miRNA, EVs, autoantibodies, the cancer microbiome and metabolomics, are rapidly emerging and opening doors for entirely new approaches to OC diagnostics and advances in imaging technologies hold promise for improving sensitivity and accuracy for identifying low-volume disease.

A key aspect for successfully implementing OC screening will be to further expand genetic testing in order to accurately and efficiently identify at-risk populations, who are expected to be the first to benefit from new screening approaches. Toward this end, risk prediction models that take into account genetic, familial, and epidemiological factors (such as the BOADICEA algorithm) continue to be refined and will undoubtedly improve targeted screening and diagnostic approaches, as well as refine clinical decision making for at-risk individuals, guiding them toward risk-reducing options such as RRSO, or for monitoring of biomarkers associated with HGSC development.

While further development and validation of the plethora of emerging biomarkers will undoubtedly take time, the failure of CA125- and TVU-based screening trials has led to a renewed emphasis on improving clinical trial design to more efficiently identify approaches with the potential to effectively reduce ovarian cancer mortality [287]. As such, the development of methods specifically designed for detecting early-stage disease and even precursor STIC lesions through minimally invasive means would be the most impactful for patients. Progress toward this goal will likely be driven by ongoing advancements in NGS, spatial transcriptomics and single-cell methods, which are likely to lead to the identification of highly specific early-stage biomarkers. While there remains much work to be accomplished for ovarian cancer screening and early diagnosis, the wealth of promising new approaches highlighted in this review may very well improve patient outcomes in the near future.

## Figures and Tables

**Table 1:** Select biomarker screening assays for the detection of epithelial ovarian cancer average specificity and sensitivity were calculated from studies with at least 250 subjects.

Test Name	Marker(s)	Modality	Potential Clinical Application(s)	Average Sens./Spec. (%)	Reference(s)
N/A	CA125	Protein	Preoperative diagnostic, Prognostic (FDA cleared)	83.7/86.0	[66,67,68]
ROMA^®^	CA125, HE4, and Menopause Status	Protein	Preoperative diagnostic, Prognostic (FDA cleared)	85.3/80.9	[68,69,70,71,72,73,74,75,76,77,78]
CPH-I	CA125, HE4, and Age	Protein	Preoperative diagnostic, Prognostic	82.2/78.9	[68,70,71,73,76,77,79]
OVA1^®^	CA125, ApoA-1, TTR, TF, and B2M	Protein	Preoperative diagnostic, Prognostic (FDA cleared)	87.7/52.6	[80,81,82,83,84,85]
Overa^®^	CA125, ApoA-1, TF, HE4, and FSH	Protein	Preoperative diagnostic, Prognostic (FDA cleared)	91.1/67.6	[86,87]
N/A	CA125, CA 15-3, CA72-4, and MCSF	Protein	Diagnostic	69.5/98.0	[88,89]
PapSEEK	ctDNA mutations	Genetic	Diagnostic, Prognostic	63.0/99.9	[90]
N/A	*OPCML* hypermethylation	Epigenetic	Screening, Diagnostic, and Prognostic	90.1/91.8	[91,92]
N/A	*ZNF154* hypermethylation	Epigenetic	Diagnostic	67.6/93.5	[93,94,95]
CancerSEEK	ctDNA mutations and glycoproteins	Pan-cancer	Screening, Diagnostic, and Prognostic	96.0/99.0	[96]
N/A	miR-200(a/b/c) + miR-320 + miR-141, among others	MicroRNA	Diagnostic, Prognostic	85.3/96.0	[97,98,99,100,101]
N/A	VLDL, LDL, lysoPC, valine, alanine, and ceramides	Metabolite	Prognostic	78.9/93.2	[102,103,104,105]

CA125, cancer antigen 125; ApoA-1, apolipoprotein A1; TTR, transthyretin; TF, transferrin; B2M, beta-2-microglobulin; HE4, human epididymis protein 4; FSH, follicle stimulating hormone; CA72-4, cancer antigen 72-4; CA 15-3, cancer antigen 15-3; MCSF, macrophage colony stimulating factor; VLDL, very low-density lipids; LDL, low-density lipids; LysoPC, Lysophosphatidylcholines; ctDNA; circulating tumor DNA.

## Supplementary Materials

The following are available online at https://www.mdpi.com/article/10.3390/cancers14122885/s1, Supplemental Table S1: Detailed table describing performance characteristics, modality, and biomarkers, from proteomic-based and preoperative approaches. Supplemental Table S2: Detailed table describing performance characteristics, modality, and biomarkers, from imaging-based approaches. Supplemental Table S3: Detailed table describing performance characteristics, modality, and biomarkers, from genetic, epigenetic and pan-cancer-based approaches. Supplemental Table S4: Detailed table describing performance characteristics, modality, and biomarkers, from emerging diagnostic and screening approaches.

## Appendix A

In order to improve patient outcomes, detection of OC should ideally be carried out well before malignant and/or metastatic transformations occur when precursor lesions and borderline tumors are in the beginning stages of development. As such, determining which factors place a woman at an increased risk of developing OC should be a priority for any screening efforts. Women with a high predisposition to ovarian cancer development are expected to particularly benefit from early surveillance and potentially risk-reducing interventions, such as risk-reducing salpingo-oophorectomy (RRSO), which can be performed when a woman’s wish of childbearing is complete. There are ongoing efforts from various medical institutions to identify the most relevant risk factors influencing cancer risk, such as a woman’s genetics, reproductive history, familial background, lifestyle, and/or other clinical features.

The most notable effort toward establishing a comprehensive risk predictive model was the Breast and Ovarian Analysis of Disease Incidence and Carrier Estimation Algorithm (BOADICEA). BOADICEA is a complex machine learning analysis tool used to compute the *BRCA1/2* mutation carrier probabilities and age-specific risks for both ovarian and breast cancers, based upon large European population-based studies of families and individuals with breast and ovarian cancer [288]. The BOADICEA algorithm has now been validated in large cohorts of women and their families in the United Kingdom [289] and the Netherlands [290] and is currently recommended as a risk assessment tool by the UK National Institute for Health and Care Excellence [291]. A second neural network model was developed by a group at Yale University, and was constructed from personal health data mined from the National Health Interview Survey [292] and from the Prostate, Lung, Colorectal and Ovarian Cancer (PLCO) Screening Trial [41]. Their model was able to characterize ovarian cancer risk into three categories: low, medium, and high-risk with an exhibited AUC of 0.71, indicating good discriminatory accuracy [293]. When implemented properly into the clinical setting, risk stratification using these “big-data” approaches can be applied to improve patient care by better identifying those factors that significantly influence a patient’s predisposition for cancer development. Discussed below are some of the most common factors that place women at a higher risk of developing OC.

### Appendix A.1. Genetic Risk Factors

It is well documented that a family history of OC places a woman at a substantially higher risk; women with an affected family member are about three times as likely to be diagnosed with ovarian cancer than those without (Odds Ratio [OR] = 3.1, 95% confidence interval [CI] = 2.6–3.7) [294]. This is likely driven by familial germline passage of genetic mutations associated with homology-directed DNA repair mechanisms such as *BRCA1/2*. The Ashkenazi Jewish population is at an exceptionally high risk of developing OC as the incidence of *BRCA1/2* mutations in this population of women is approximately 1:40, compared to about 1:500 for the general population [295]. Indeed, genetic testing for high and moderate penetrance genes to identify at-risk patients can be of benefit to individuals by directing them to appropriate preventative steps, more frequent monitoring, and/or personalized treatment options. As such, the FDA has approved genetic-based diagnostic tests such as MyChoice^®^ HRD CDx (PMA P190014) [296] launched by Myriad Genetic Laboratories, Inc., Salt Lake City, UT, USA, which is an NGS based test on formalin-fixed ovarian tissue for identifying patients who are positive for homologous recombination deficiencies (*BRCA1/2*) and would benefit from treatment with PARP inhibitors. While germline mutations in these genes are highly associated with cancer development, they only appear in about 15% of all OC cases [297]. Furthermore, in women with no family history, somatic mutations or epigenetic silencing in these driver genes present an equally high risk for developing the disease [20]. Regardless of mutational inheritance status, it was shown that women with mutations in *BRCA1* have a 35–70 fold increased risk and those with *BRCA2* mutations have a 10–30-fold increased risk [298,299] compared to the general population. Combined, the presence of germline and somatic *BRCA1/2* genetic mutations in HGSC represents only about one-fourth of the cases and targeting early screening to just this population would only moderately improve outcomes for OC patients as a whole.

### Appendix A.2. Reproductive Risk Factors

The frequency and the total number of ovulation cycles are positively associated with a woman’s risk for developing ovarian cancer. During ovulation, the processes involved in follicular rupture and release of the oocyte from the ovary can promote malignant transformation of fallopian tube epithelia. When an egg ruptures from the follicle, tissue-resident immune cells undergo transient activation through cytokine and chemokine release, resulting in a temporary inflammatory-like microenvironment [300]. This inflammatory response, repeated multiple times over the course of a woman’s fertile window, has the potential to promote the malignant epithelial cell transformation of tubal cells. Consequently, the majority of HGSC diagnoses are in women above the age of 50; The American Cancer Society reports that half of ovarian cancer cases are in women who are 63 years or older. Epidemiologic studies have shown that for women who are less than 50 years old, their risk of developing OC increases by 2% for every additional year of age, and for women who are greater than or equal to 50 years old, their risk increases by 11% for every additional year [301]. In addition, age of menarche and age of menopause are both associated with an increased risk of developing OC. It is clear that ovulation plays an important role in a woman’s risk of developing ovarian cancer, as certain factors modulating the frequency and occurrence of ovulation, such as birth control and the number of pregnancies [298,302,303] were shown to significantly lower a woman’s risk of developing OC. In fact, the use of oral contraceptives for greater than 5 years is associated with a 30 to 50% risk reduction for OC development [304], and therefore birth control pills were proposed as a first-line preventative that can be easily prescribed to the general population.

### Appendix A.3. Dietary and Lifestyle Risk Factors

Diet has also been shown to be a risk factor for OC development. Studies have illustrated that increasing intake of dietary fiber [305,306] or Vitamin D [307] negatively correlates with ovarian cancer risk. In a large case-control study performed in China, Zheng et al., found that an increased intake in non-salted vegetables (OR = 0.24, 95% CI = 0.1–0.5) and fruits (OR = 0.36, 95% CI = 0.2–0.7) was negatively associated with OC risk [308]. Alternatively, intake of saturated fats [302,308] and patient obesity [309,310] are positively correlated with risk. A meta-analysis study looking at the correlation between women who have diabetes mellitus and ovarian cancer found that women with diabetes are 17% more likely to develop cancer (OR = 1.16, 95% CI = 1.01–1.33) [311]. Furthermore, in a large retrospective cohort study, Akhavan, et al. illustrated that OC patients that present with diabetes mellitus had significantly shorter overall survival compared to those who did not (OR = 4.76, 95% CI = 2.99–7.59) [312]. Conversely, those who had a higher “healthy lifestyle index” (defined by smoking status, height, weight, physical activity, diet and alcohol consumption) saw a longer overall survival compared to those with a lower index (OR = 0.79, 95% CI = 0.59–1.04) [313]. While it is clear that an unhealthy lifestyle can put women at greater risk of developing OC, there is little correlation between ovarian cancer risk and obesity. In an analysis of the Kaiser Permanente Northern California (KPNC) primary invasive epithelial ovarian cancer cohort, only 36% of patients were reported to be obese (BMI < 30 kg m^−2^) [314]. Consequently, early screening focused on obese and diabetic women would lead to diminished test specificity, as many of these women would likely be sent for needless follow-up.
